# Disordered proteins mitigate the temperature dependence of site-specific binding free energies

**DOI:** 10.1016/j.jbc.2023.102984

**Published:** 2023-02-03

**Authors:** Joseph F. Thole, Christopher A. Waudby, Gary J. Pielak

**Affiliations:** 1 Department of Chemistry, UNC-Chapel Hill, Chapel Hill, North Carolina, USA; 2 Molecular and Cellular Biophysics Program, UNC-Chapel Hill, Chapel Hill, North Carolina, USA; 3 School of Pharmacy, University College London, London, United Kingdom; 4 Department of Biochemistry & Biophysics, UNC-Chapel Hill, Chapel Hill, North Carolina, USA; 5 Lineberger Cancer Center, UNC-Chapel Hill, Chapel Hill, North Carolina, USA; 6 Integrative Program for Biological and Genome Sciences, UNC - Chapel Hill, Chapel Hill, North Carolina, USA

**Keywords:** intrinsically disordered protein, protein–protein interaction, Src homology 3 domain, biophysics, thermodynamics, Drk, Downstream of receptor kinase, IDP, intrinsically disordered protein, IDR, intrinsically disordered region, ITC, isothermal titration calorimetry, LB, Lennox broth, MBD, maltose-binding domain, SH3, Src homology 3, Sos, Son of sevenless

## Abstract

Biophysical characterization of protein–protein interactions involving disordered proteins is challenging. A common simplification is to measure the thermodynamics and kinetics of disordered site binding using peptides containing only the minimum residues necessary. We should not assume, however, that these few residues tell the whole story. Son of sevenless, a multidomain signaling protein from *Drosophila melanogaster*, is critical to the mitogen-activated protein kinase pathway, passing an external signal to Ras, which leads to cellular responses. The disordered 55 kDa C-terminal domain of Son of sevenless is an autoinhibitor that blocks guanidine exchange factor activity. Activation requires another protein, Downstream of receptor kinase (Drk), which contains two Src homology 3 domains. Here, we utilized NMR spectroscopy and isothermal titration calorimetry to quantify the thermodynamics and kinetics of the N-terminal Src homology 3 domain binding to the strongest sites incorporated into the flanking disordered sequences. Comparing these results to those for isolated peptides provides information about how the larger domain affects binding. The affinities of sites on the disordered domain are like those of the peptides at low temperatures but less sensitive to temperature. Our results, combined with observations showing that intrinsically disordered proteins become more compact with increasing temperature, suggest a mechanism for this effect.

Approximately 40% of the eukaryotic proteome comprises intrinsically disordered proteins (IDPs) or intrinsically disordered regions (IDRs) (1, 2), many of which function in signal transduction (3, 4). Their mechanisms of interaction and binding vary, ranging from folding upon binding (5) to “fuzzy” interactions (6). Protein–protein complexes where at least one partner is disordered tend to be less stable than complexes formed between folded species (7), but exceptions exist (8). Disordered interactions are enriched in signaling pathways because they are highly tunable so outcomes can be altered in response to external stimuli and feedback (3, 4, 9, 10, 11). Nevertheless, we lack a general understanding of IDP–IDR function and behavior because of their diverse mechanisms and because the proteins often have challenging physical properties (*e.g.*, they tend to aggregate and phase separate).

The mitogen-activated protein kinase pathway is a well-conserved signaling regime that allows cells to differentiate, divide, respond to stress, and undergo apoptosis (12, 13). In *Drosophila melanogaster*, extracellular signals (Spitz, Trunk, Bride of sevenless, etc.) begin the signaling cascade, leading to the activation of Son of sevenless (Sos) (14, 15, 16).

Sos is a 178 kDa multidomain protein with guanine nucleotide exchange factor activity that further stimulates the GTPase, Ras (Fig. 1*A*). The human homolog, SOS1, undergoes two forms of autoinhibition. The N-terminal Dbl- and Pleckstrin-homology domains require interaction with phosphatidylinositol 4,5-bisphosphate to recruit Ras, ensuring localization to the membrane (17, 18, 19). At the C terminus, a 55 kDa proline-rich disordered domain binds the Src homology 3 (SH3) domain of Downstream of receptor kinase (Drk)/GRB2 (*D. melanogaster*/human), which is recruited by an activated receptor tyrosine kinase *via* a phosphorylated cytosolic Tyr (14, 20, 21). The inhibitory mechanism of the C-terminal domain is unknown.

**Figure 1:**
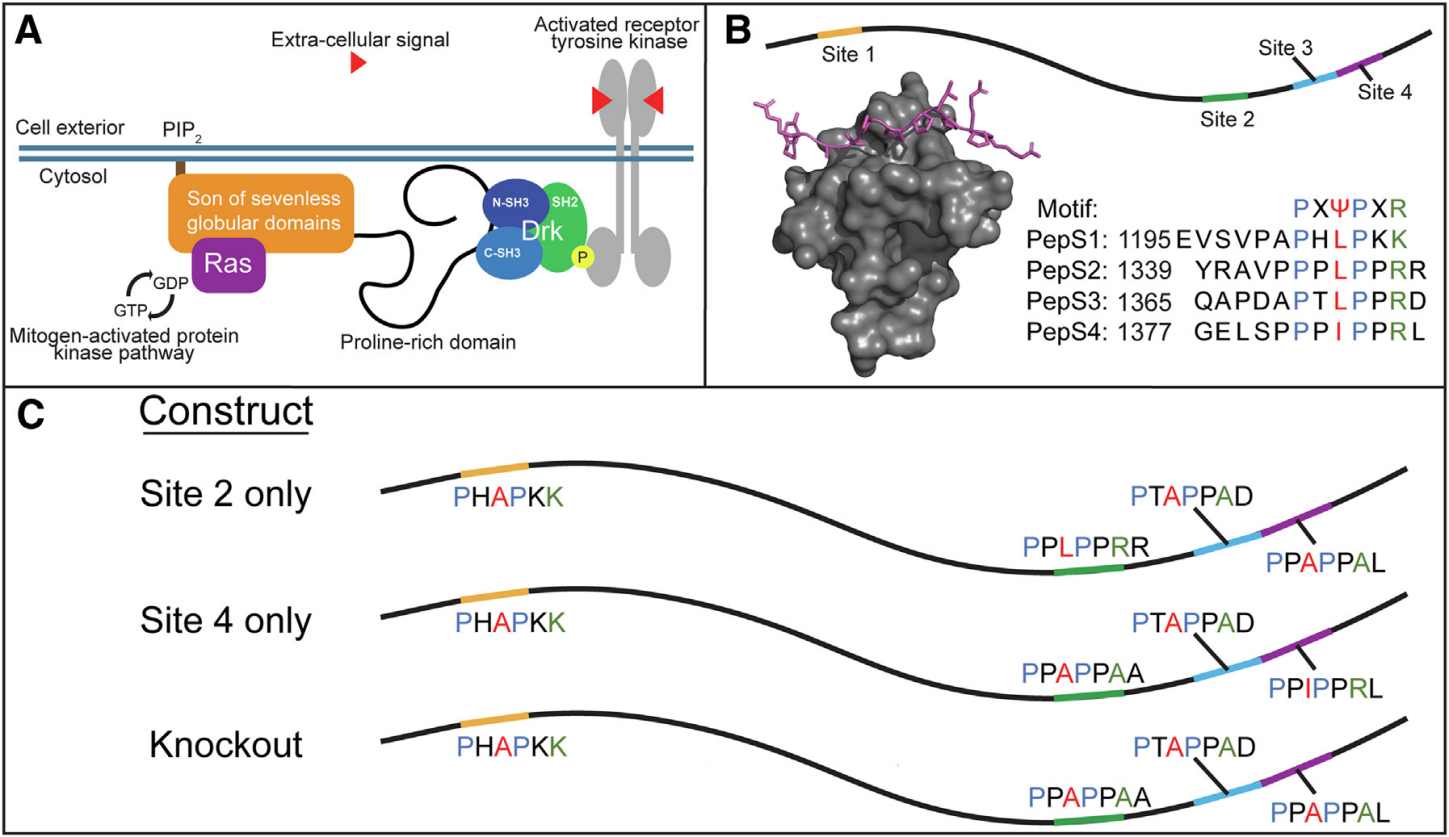
**Sos (Son of sevenless)–Src homology 3 (SH3) binding.***A,* simplified model of Ras activation by Sos. *B,* representation of SH3-binding sites on Sos disordered C-terminal tail. Simulation of PepS2 binding to SH3 domain (70). Sequence alignment of binding motif with Sos-binding sites (21, 24). *C,* model of site 2, site 4, and knockout constructs with conserved amino acid positions highlighted at each site (as in *B*).

Sos proline-rich sequences form type-II left-handed helices and bind SH3 using the P-X-Ψ-P-X-R motif, where Ψ is Leu/Ile/Val and R is Arg or Lys (21, 22, 23). Similar to SOS1 (21, 23), Sos has at least four binding sites: sites 2 and 4 have low micromolar affinities, whereas sites 1 and 3 have high micromolar or millimolar affinities (Fig. 1*B*) (24). Until now, efforts to quantify these affinities, like many efforts involving a disordered protein, focused only on peptides containing the key residues and immediately flanking sequences. This reasonable choice simplifies analysis, yet we know that residues beyond the binding site modulate affinity (25). Our goal was to determine how the context of the disordered region affects SH3 binding. We measured SH3 binding to the Sos site in short peptide form versus the extended disordered domain.

We used techniques known to work for IDPs and IDRs—isothermal titration calorimetry (ITC) and NMR spectroscopy—to distinguish binding of SH3 to individual sites from nonspecific or weak binding (24, 26, 27) and focused on sites 2 and 4, which interact most strongly with SH3 as peptides. We found that the single-site affinities are comparable to those for the peptides, but binding to the protein is less temperature sensitive because of enthalpy–entropy compensation (28). As our approach is applicable to other disordered proteins, we expect that it may help to unravel both kinetic and thermodynamic aspects of other IDP interactions.

## Results

Multitemperature NMR datasets for the site 4 peptide binding and a one-temperature dataset for the site 2 peptide binding are published (24). We began by completing ^19^F-NMR titrations of SH3 (using a single ^19^F atom on W36 *via* 5-fluoroindole labeling (29)) with the site 2 peptide. We triplicated a multitemperature dataset using methods we established (24, 30). Briefly, spectra from a single titration series were fit to a two-state binding model using lineshape analysis. This approach allows us to estimate affinity, *k*_on_, and *k*_off_ by simulating the spectra series using least-squares fitting. Each temperature (4.2–45 °C) was fit independently. The fitted parameter estimates were then bootstrapped (n = 1000) and fit to the van’t Hoff equation to estimate ${\Delta H}_{D}^{{}^{\circ}\prime }$ and ${T\Delta S}_{D}^{{}^{\circ}\prime }$ and to the Eyring equation to estimate ${\Delta H}_{A}^{{}^{\circ}\prime ‡}$, $T{\Delta S}_{A}^{{}^{\circ}\prime ‡}$, ${\Delta H}_{D}^{{}^{\circ}\prime ‡}$, and $T{\Delta S}_{D}^{{}^{\circ}\prime ‡}$. Uncertainties are the standard deviations of the bootstrapped parameters. For site 2 peptide binding, the equilibrium is enthalpically favored and entropically disfavored (Table 1 and Figs. S1 and S2). Kinetically, both association and dissociation are enthalpically disfavored and entropically favored. There are no important differences between the thermodynamics or kinetics of SH3 binding to the site 2 and site 4 peptides.

**Table 1:** Comparison of NMR parameters and dissociation free energies of Sos site peptides and single sites on Sos protein as a function of temperature

Construct	Temperature (°C)	*K*_*D*_ (μM)	${\Delta G}_{D}^{o’}$ (kcal/mol)	*k*_on_ (10^8^ M^−1^ s^−1^)	${\Delta G}_{A}^{o’‡}$ (kcal/mol)	*k*_off_ (10^3^ s^−1^)	${\Delta G}_{D}^{o’‡}$ (kcal/mol)
Peptide 2^a^	4.2	9 ± 2	6.5 ± 0.3	0.6 ± 0.2	6.3 ± 0.3	0.486 ± 0.003	12.825 ± 0.007
	15	17 ± 2	6.3 ± 0.1	0.7 ± 0.1	6.5 ± 0.1	1.23 ± 0.02	12.77 ± 0.02
	25	33 ± 3	6.12 ± 0.09	1.1 ± 0.1	6.48 ± 0.09	3.55 ± 0.05	12.61 ± 0.01
	35	59 ± 4	5.96 ± 0.07	1.5 ± 0.1	6.53 ± 0.07	8.88 ± 0.05	12.492 ± 0.006
	45	110 ± 20	6.1 ± 0.1	1.7 ± 0.3	6.7 ± 0.2	17 ± 2	12.5 ± 0.1
Peptide 4^a^^,^^b^	5	20 ± 10	6.2 ± 0.2	0.2 ± 0.06	7.0 ± 0.1	0.2 ± 0.07	13.1 ± 0.1
	15	40 ± 10	6.0 ± 0.3	0.4 ± 0.2	7.0 ± 0.3	0.9 ± 0.1	13.0 ± 0.1
	25	60 ± 10	5.8 ± 0.1	0.6 ± 0.1	7.0 ± 0.1	2.9 ± 0.2	12.7 ± 0.1
	35	110 ± 10	5.6 ± 0.1	0.57 ± 0.08	7.1 ± 0.1	5.8 ± 0.3	12.8 ± 0.1
	45	210 ± 30	5.4 ± 0.1	1.1 ± 0.1	7.0 ± 0.1	23 ± 3	12.2 ± 0.1
Sos site 4^c^	4.2	3.0 ± 0.2	7.01 ± 0.07	0.31 ± 0.02	6.69 ± 0.07	0.093 ± 0.002	13.70 ± 0.02
	15	4.5 ± 0.2	7.05 ± 0.04	0.56 ± 0.03	6.64 ± 0.06	0.25 ± 0.09	13.69 ± 0.04
	25	6.4 ± 0.2	7.09 ± 0.03	0.97 ± 0.06	6.56 ± 0.06	0.62 ± 0.03	13.64 ± 0.05
	35	9.7 ± 0.2	7.07 ± 0.02	1.29 ± 0.05	6.62 ± 0.04	1.25 ± 0.04	13.69 ± 0.03
	45	17.8 ± 0.5	6.91 ± 0.03	2.5 ± 0.1	6.45 ± 0.05	4.4 ± 0.2	13.36 ± 0.05

a Uncertainties from triplicate analysis.

b Published (24).

c Uncertainties from bootstrap analysis of a single measurement.

Initial efforts to purify the disordered Sos protein were unsuccessful because of nonspecific hydrophobic interactions that caused Sos to coelute with contaminants. Adding 10% v/v propylene glycol to the buffer solved the problem. The conserved leucines/isoleucines and arginines in the Sos-binding sites are critical to binding (21, 31). To focus on the higher affinity sites, 2 and 4, we used alanine substitutions to weaken the lower affinity sites, 1 and 3. The same approach was used at the stronger sites, 2 or 4, to focus on a single binding site (Fig. 1*C*). We attempted to quantify the affinities of site 4-only constructs using ^19^F lineshape analysis as per our peptide data (Fig. S3*A*) but obtained poor fits. This failure probably arises from weak and nonspecific ^19^F-labeled SH3 binding (see Discussion section) across the entire protein, so we turned to ITC.

**Figure 2:**
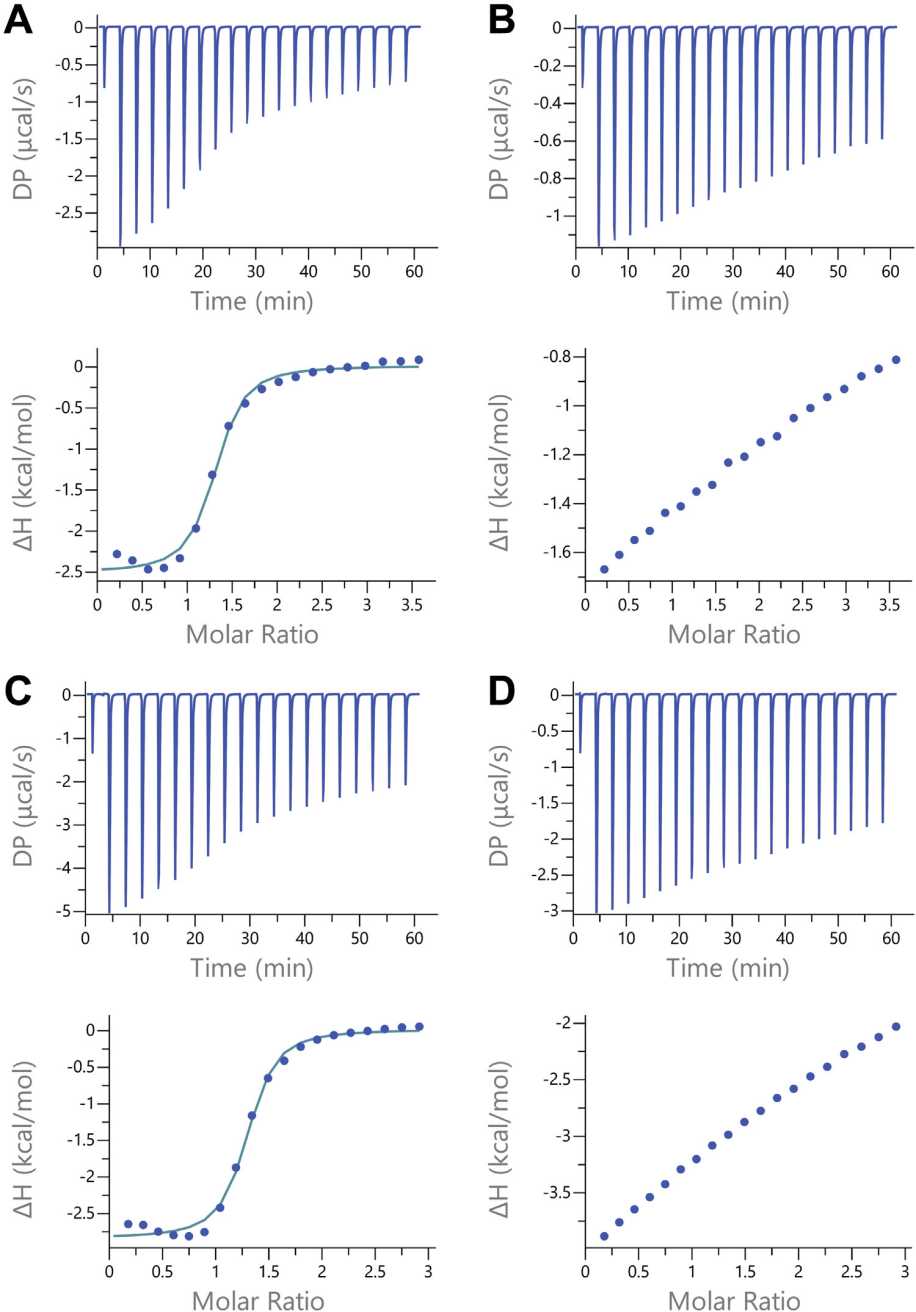
**ITC analysis of Sos–SH3 binding at 4.2 °C.***A,* Sos site 2. *B,* Sos site 2-matched knockout. *C,* Sos site 4. *D,* Sos site 4-matched knockout. *A* and *B* were performed with 229 μM Sos and 4 mM SH3. *C* and *D* were performed with 250 μM Sos and 3.56 mM SH3. ITC, isothermal titration calorimetry; SH3, Src homology 3; Sos, Son of sevenless.

ITC measures the change in heat associated with binding, and when an experiment is well designed, can quantify the stoichiometry, ${\Delta G}_{D}^{{}^{\circ}\prime }$, ${\Delta H}_{D}^{{}^{\circ}\prime }$, and ${T\Delta S}_{D}^{{}^{\circ}\prime }$. A careful approach requires a “c-value” $\left(c=\frac{n∙\left[M\right]}{K_{D}}\right)$ between 5 and 500, where n is the stoichiometry and M is the protein concentration (27, 32, 33). ITC measurements of the site 2- and site 4-only constructs showed that the interaction remained unsaturated even after adding >3 mole equivalents, showing that the stoichiometry is >1, which we suspect is a combination of the single strong site and weak and nonspecific interactions observed during purification and in the ^19^F NMR experiments (Fig. 2). To isolate the signal from individual sites, we needed to overcome the heat signal from nonspecific binding, leading us to design a knockout construct in which all four specific sites were abolished by amino acid changes to alanine (Fig. 1*C*). We discuss the effectiveness of knockouts in the supporting information (Fig. S4 and Table S1). The knockout measurements showed injection heats of the same magnitude as the heat at the end of the titration, where we expected heats from an ideal single-site construct to approach baseline levels (Fig. 2*C*). We could not reliably fit the knockout to a binding isotherm, as the c-value was approximately 0.1 (33); however, the alignment of the single-site construct background heats and the knockout suggests that our approach accurately represents the nonspecific component of the interaction.

We used the knockout controls, prepared side by side with the construct of interest, to subtract nonspecific binding. This approach yielded reasonable fits (Fig. 2 and Table 2) and stoichiometries of one SH3 per Sos. Site 2 has an affinity of 4 ± 1 μM and an $\Delta H_{D}^{{}^{\circ}\prime }$ of 3.2 ± 0.1 kcal/mol at 4.2 °C. Site 4 has an affinity of 3 ± 1 μM and an $\Delta H_{D}^{{}^{\circ}\prime }$ of 2.8 ± 0.1 kcal/mol at 4.2 °C. At 35 °C, both sites have affinities of 14 ± 3 μM and $\Delta H_{D}^{{}^{\circ}\prime }$ values of 8.4 ± 0.3 kcal/mol, indicating a positive $\Delta C_{P,D}^{{}^{\circ}\prime }$ (Table 2).

**Table 2:** Parameter estimates for Sos site 2 and Sos site 4 from ITC measurements

Site	Temperature (°C)	N^a^	*K*_*D*_^a^ (μM)	${\Delta G}_{D}^{{}^{\circ}\prime }$^b^ (kcal/mol)	${\Delta H}_{D}^{{}^{\circ}\prime }$^a^ (kcal/mol)	T${\Delta S}_{D}^{{}^{\circ}\prime }$^b^ (kcal/mol)
2	4.2	1.24 ± 0.03	5 ± 2	6.76 ± 0.03	2.5 ± 0.1	−4.25 ± 0.07
	35	1.21 ± 0.01	14 ± 1	6.86 ± 0.01	8.9 ± 0.1	2.1 ± 0.1
4	4.2	1.23 ± 0.02	3 ± 1	6.95 ± 0.02	2.9 ± 0.1	−4.10 ± 0.03
	35	1.27 ± 0.02	14 ± 3	6.84 ± 0.02	8.4 ± 0.3	1.5 ± 0.1

a Uncertanties derived from error propagation of three fits.

b Uncertainties are the standard deviation of three estimates.

The subtraction does not perfectly account for the nonrandom order in which SH3 binds Sos. In early stages of the titration, we expect SH3 to prefer the higher-affinity site compared with the weaker and nonspecific sites in the knockout. This effect is observed as an initial oversubtraction (<0.5 mole ratio). Toward the end of the titration, as the stronger binding site becomes fully occupied, more nonspecific binding occurs, resulting in better agreement between the data and fit. Importantly, when we compare this approach to direct measurements of site 4 binding *via* NMR (described later), the two methods yield the same information. In essence, our approach is effective because the difference in affinities is large, allowing nonspecific binding to be treated as random events. If the affinities were more similar, our approach would fail. Another advantage to this method is that the knockout subtraction inherently accounts for the heat of ligand dilution.

Given these ITC data, we reevaluated the ^19^F NMR measurements of SH3 binding to Sos. We tried to fit the data to a bidentate model in which the Sos ligand contains two binding sites, representing a single high-affinity binding site, constrained by ITC measurements, and a second site representing the combination of weak and nonspecific binding (observed *via* ITC in the knockout construct). However, we found that this model was also unable to generate acceptable fits (Fig. S3 and Table S2) consistent across all observed temperatures. We conclude that the interaction with Sos, observed from the perspective of SH3, can occur *via* too many weak or nonspecific sites to be accounted for by a simple two-state or bidentate binding model.

**Figure 3:**
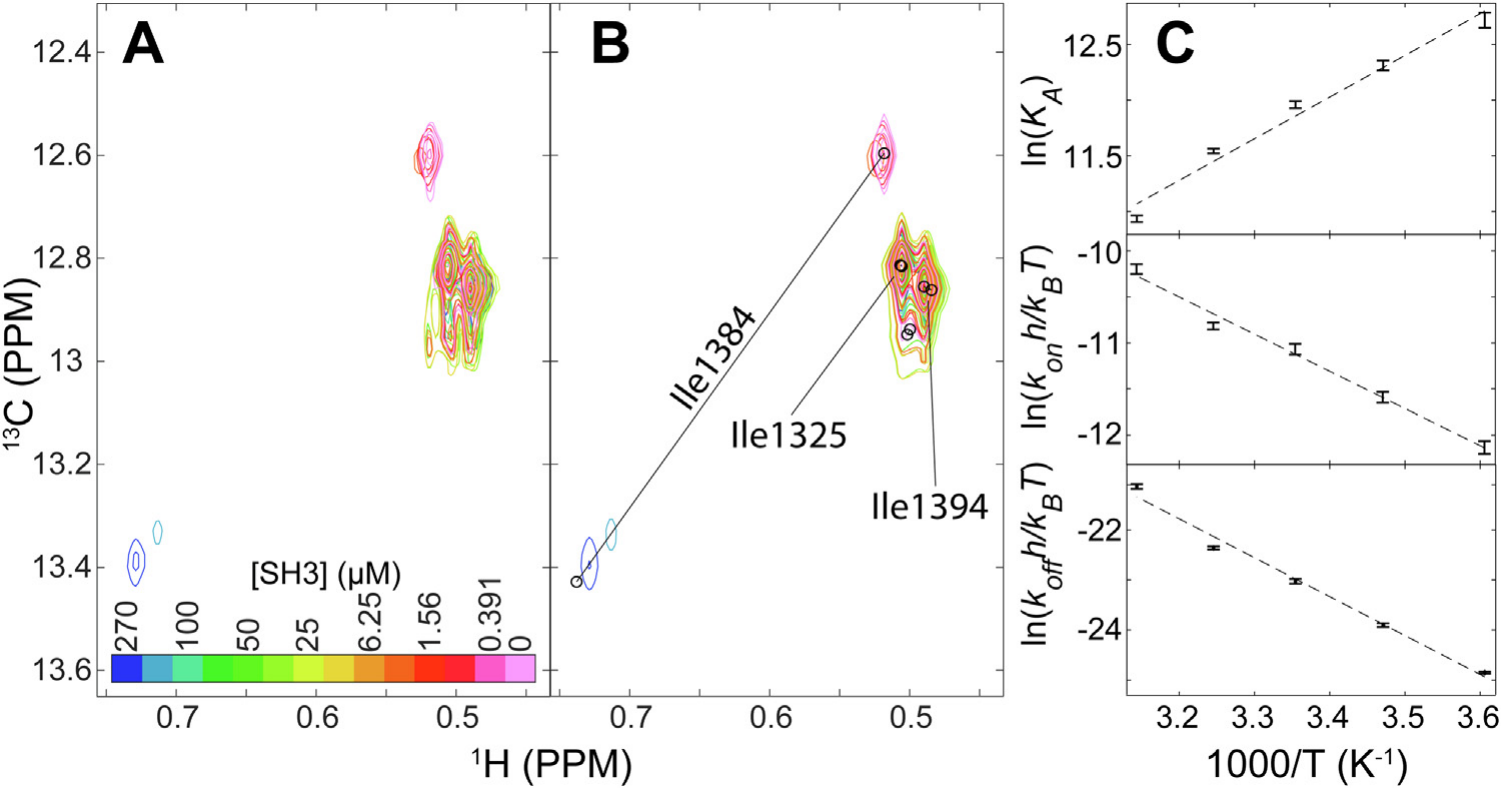
^**1**^**H–**^**13**^**C HMQC titration of Sos site 4 at 35 °C.***A,* spectra. *B,* simulated spectra from TITAN fits. *C,* van’t Hoff and Eyring analyses of ^1^H–^13^C HMQC titrations. Measurements were made with a constant concentration of 51.5 μM Sos site 4, with titration points signified by crosspeaks of different colors. SH3 concentrations are 270, 122, 100, 75.0, 50.0, 37.5, 25.0, 12.5, 6.25, 3.13, 1.65, 0.781, 0.391, and 0 μM. HMQC, heteronuclear multiple quantum correlation; Sos, Son of sevenless.

As an alternative, we measured binding of Sos and SH3 from the opposite perspective, that is, by monitoring the interaction *via* Sos by moving the NMR-active nuclei from SH3 to Sos. Specifically, we isotopically enriched (^13^C-δ1 methyl) isoleucines in the site 4 protein construct. This construct has three isoleucines: I1384 is in site 4; I1394 is adjacent to site 4; and I1325 is likely distant from site 4. Assignments were made *via* mutagenesis (Supplemental Material, Fig. S5). As expected, upon titration with SH3 (Fig. 3), the I1384 crosspeak is most sensitive in terms of chemical shift changes, the I1394 crosspeak undergoes some shift, and the I1325 crosspeak does not change (Fig. 3*A*). High-quality fits were obtained using two-dimensional lineshape analysis (Figs. S6–S10), yielding affinities (Table 1) similar to those from ITC (Table 2), and site 4 peptide, but only at low temperatures (Table 1) (34). The exchange rates from the ^1^H–^13^C data parallel those from the stronger ^19^F fitted site (Tables 1 and S2) indicating that the kinetics are similar whether we monitor binding *via* SH3 or Sos site 4 despite the uncertainties in the ^19^F fit.

In terms of equilibrium binding, linear fitting of van’t Hoff plots yielded a ${\Delta H}_{D}^{{}^{\circ}\prime }$ of 7 ± 2 kcal/mol and a $T{\Delta S}_{D}^{{}^{\circ}\prime }$ of 0 ± 2 kcal/mol at 298 K. Although we know that ${\Delta C}_{p,D}^{{}^{\circ}\prime }$ is positive from the change in ${\Delta H}_{D}^{{}^{\circ}\prime }$ with temperature (Table 2) and because the van’t Hoff data are curved (Figs. 3*C* and S11), its magnitude is small, making a linear fit reasonable. In summary, we find that binding is driven by a decrease in enthalpy, and the entropic change is small.

In summary, the equilibrium results from ITC and NMR for SH3–Sos binding agree with one another. The equilibrium enthalpic and entropic contributions to binding are indistinguishable between methods. The small differences in affinities may arise from the use of D_2_O (35) in the NMR experiments or small differences in protein concentration estimates.

Turning to kinetics, we used linear Eyring analysis of *k*_on_ and *k*_off_ to characterize the activation parameters for the interaction of Sos with SH3. We obtained a ${\Delta H}_{A}^{{}^{\circ}\prime ‡}$ of 7 ± 1 kcal/mol and a $T{\Delta S}_{A}^{{}^{\circ}\prime ‡}$ of 1 ± 1 at 298 K. The activation enthalpy of dissociation, ${\Delta H}_{D}^{{}^{\circ}\prime ‡}$, was 14 ± 1 kcal/mol with a $T{\Delta S}_{D}^{{}^{\circ}\prime ‡}$ (at 298 K) of 1 ± 2. Thus, the reaction in both directions shows little to no entropic contribution but must overcome an enthalpic barrier to associate and a higher enthalpic barrier to dissociate.

## Discussion

The affinities of site 4 for SH3 in the peptide and the IDP are similar at low temperatures and higher at high temperatures. Further analysis reveals two important changes compared with the peptide data. One difference concerns the difference in heat capacity between the reactants and products, and the other focuses on the entropy change for the reaction.

${\Delta H}_{D}^{{}^{\circ}\prime }$ of SH3–peptide binding remains favorable at all temperatures and drives binding, in line with other studies of SH3–peptide interactions (21, 24, 36). Also, the peptide data are well fit by linear van’t Hoff analysis (Fig. S3), suggesting that $\Delta C_{P,D}^{{}^{\circ}\prime }$ is small. Turning to the disordered protein, although the data are too sparse for quantification, we observe a positive $\Delta C_{P,D}^{{}^{\circ}\prime }$ because the ${\Delta H}_{D}^{{}^{\circ}\prime }$ becomes more favorable with increasing temperature (Table 1).

Next we considered the entropic contribution to binding. At equilibrium, the change is favorable at low temperatures and becomes less favorable at higher temperatures (Table 2) but is always unfavorable for the peptides (Fig. S1). If the only factor was the mixing of SH3 and Sos, we expect unfavorable entropic contributions at all temperatures, as observed for SH3–peptide binding (14, 21, 24, 37, 38, 39). Although interpretation of entropic contributions is problematic, our observation is consistent with the temperature dependence of IDP hydrodynamic radii, in that IDPs become more compact at higher temperatures (40, 41, 42, 43, 44, 45, 46). Explanations for this observation suggest the release of water because the strength of the hydrophobic effect increases with temperature up to 110 °C, after which the effect is expected to cause collapse (44, 47, 48, 49, 50). At low temperatures, we would expect the IDP to be more solvated, and upon binding, will release water near the binding interface. This release would result in a favorable change in entropy that pays the cost of demixing. At higher temperatures, the IDP has already undergone compaction, leaving less water to be released, and there is less compensation for the penalty of demixing.

An alternative explanation is that at lower temperatures, Sos possesses transient structure, and that structure is disrupted when SH3 binds. Taking the classical view of heat-induced protein denaturation (51), this structure would melt at higher temperatures, and the disordered protein would act more like the peptide. The equilibrium thermodynamics of binding at high temperatures (Table 2 and Fig. S1) are consistent with this idea, but such an analysis would require that the temperature dependence of the Sos IDR hydrodynamic radius contradict the known behavior of other IDPs (40, 41, 42, 43, 44, 45, 46).

**Figure 4:**
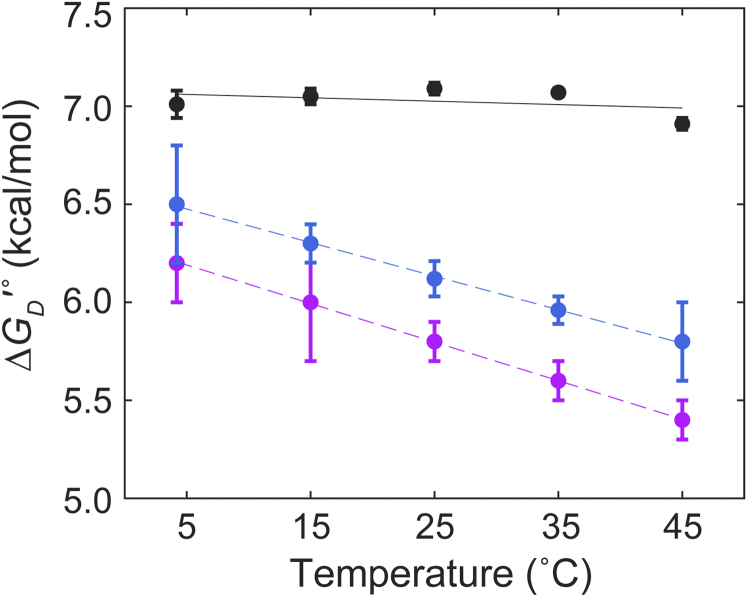
**Temperature dependence of SH3–Sos dissociation free energy.** SH3, Src homology 3; Sos, Son of sevenless.

In summary, for peptides, ${\Delta G}_{D}^{\prime {}^{\circ}}$ becomes less favorable as temperature increases but is nearly invariant for the disordered protein, at least over the temperatures examined (Fig. 4). Such a result has been reported before for an IDP (27). This compensation is potentially beneficial for cells because the invariance would require fewer regulatory mechanisms to respond to temperature changes.

Turning to kinetics, the *k*_on_ values for the peptides and the IDP are all near diffusion limited, but the presence of the IDP decreases *k*_off_. Combining this observation with the more favorable ${\Delta G}_{D}^{{}^{\circ}\prime }$ of the IDP suggests that the bound state is simply more stable because of contacts with extra residues bordering the binding site. The activation parameters, ${\Delta G}_{D}^{{}^{\circ}\prime ‡}$ and ${\Delta G}_{A}^{{}^{\circ}\prime ‡}$ for Sos site 4, do not change with temperature, limiting evaluation using Leffler-like analysis of the transition complex (52). However, a parsimonious interpretation is that the mechanism does not change over the temperature range. It is also reasonable to assume that the changes observed between the site 4 peptide and the IDP will hold with the site 2 peptide compared to the IDP, given the similarities in energetics and kinetics between site 2 and 4 peptides (Table 1) and the similarities in the IDP sites (Table 2).

In summary, we show that detailed quantification of an extended-IDP interaction is possible. Although measuring the interaction *via* the ligand (^19^F-labeled SH3) is challenging because of nonspecific binding, monitoring residues in the binding site (^13^C-enriched Sos) offers an alternative that will facilitate characterization of other binding-competent IDPs. Looking forward, more kinetic and equilibrium data on IDPs are required to develop a general view of disordered proteins. To build this view, we must combine information on specific interactions as well as weaker and nonspecific interactions that may contribute to biological outcomes and play a role in the allovalency of IDPs with multiple binding sites (53, 54, 55, 56).

Overall, interaction of the stabilized N-terminal SH3 domain of Drk with its native binding sequences is similar mechanistically if the sites are on a short peptide or in the context of the larger disordered protein. However, the additional residues in the IDP cause enthalpy–entropy compensation such that ${\Delta G}_{D}^{{}^{\circ}\prime }$ varies less with temperature compared with the peptide. We also observe that at low temperatures, binding to the IDP is entropically favorable but becomes less favorable with increasing temperature. Our experimental framework will facilitate the study of more IDP-binding systems and provide a better understanding of IDPs.

## Experimental procedures

### Construct designs

The Sos intrinsically disordered region was designed from *D.* *melanogaster* Sos (UniProt ID: P26675) and comprises residues 1177 to 1405 (Fig. S12). Constructs were cloned into pET28b plasmids with a C-terminal cysteine self-cleaving peptide (57) followed by a decahistidine tag or hexahistidine tag. We later observed that an N-terminal maltose-binding domain (MBD), followed by a tobacco etch virus cleavage site, improved the yield. To focus on the strongest sites (2 and 4), we needed to reduce binding at the weaker sites (1 and 3) but wanted to minimally perturb any structural character of the region (*i.e.*, maintain the native prolines). It was shown that in the PXΨPXR motif, the Ψ followed by R residues were the most consequential to binding (21, 31), so we used alanine substitutions for these residues to weaken the sites. Site 1 was already shown to bind with millimolar affinity (24), so the single L1203A substitution was chosen. For site 3, the L1372A and R1375A substitutions were selected. C1306S and C1357S substitutions were also introduced to prevent disulfide bond formation. The site 2 construct also contained the I1384A and K1387A substitutions that reduce binding to site 4. The site 4 construct has the L1346A, R1349A, and R1350A substitutions that reduce binding to site 2. Site 2 has two positively charged residues at the C terminus of the binding site, whereas site 4 has only one (Fig. 1). It has been shown that multiple positively charged residues can enhance binding (21), so it was necessary to substitute all of them. The knockout construct contained all the described substitutions. Following purification (described later), these constructs contain additional SMG (with MBD)- or MG (without MBD) residues at the N terminus and QSL residues at the C terminus.

### Expression and purification

Unenriched Sos was prepared as follows. The pET28b plasmid containing the construct of interest was transformed into BL21 (DE3) *Escherichia coli* cells (Thermo Fisher Scientific). A 5 ml liquid culture of 25 g/l Lennox broth (LB, 10 g/l tryptone, 5 g/l yeast extract, and 5 g/l NaCl) containing 50 mg/l kanamycin was inoculated with a single colony and shaken at 37 °C, 225 rpm (Innova I26). After >6 h, 200 ml of LB was inoculated with 200 μl of the smaller culture. The 200 ml culture was shaken overnight at 37 °C, 225 rpm. The next day, 1 l cultures were inoculated with 10 or 20 ml of the overnight culture and grown at 37 °C, 225 rpm. Isopropyl β-d-1-thiogalactopyranoside (1 ml of a 1 M solution) was added when the absorbance at 600 nm reached 0.6 (∼2 h). Cultures were then shaken overnight at 20 °C.

[^13^CH_3_-Ile]-enriched Sos was prepared as follows: a 5 ml liquid culture of 25 g/l LB and 50 mg/l kanamycin was inoculated from a single colony and shaken at 37 °C, 225 rpm. After >6 h, a 750 ml solution of 2× M9 media (100 mM Na_2_HPO_4_, 40 mM KH_2_PO_4_, 9 mM NaCl, 3 g/l glucose, 1 g/l NH_4_Cl, 0.1 mM CaCl_2_, 2 mM MgSO_4_, 10 mg/l thiamine, 10 mg/l biotin, and 50 mg/l kanamycin, pH 7.4) was inoculated with 750 μl of the smaller culture. The 750 ml culture was shaken overnight at 37 °C, 225 rpm. The next day, 1 l cultures were inoculated with 50 ml of the overnight culture and grown at 37 °C, 225 rpm. When the absorbance at 600 nm reached 0.55 (∼6 h), 60 mg/l [3,3-D_2_] ^13^C α-ketobutyric acid (58, 59) was added, and the temperature was lowered to 20 °C. After 30 min, 1 ml of 1 M isopropyl β-d-1-thiogalactopyranoside was added, and the cultures were shaken overnight at 20 °C.

Following overnight incubation, the cultures were transferred to 1 l bottles and centrifuged at 1000*g* for 30 min. The pellets were resuspended in loading buffer (15.1 mM Na_2_HPO_4_, 4.9 mM NaH_2_PO_4,_ 20 mM imidazole, 300 mM NaCl, 2 mM tris(2-carboxyethyl)phosphine hydrochloride, 10% v/v propylene glycol, pH 8.0), and protease inhibitors were added (Sigma–Aldrich; P2714). Cells were lysed using a sonic dismembrator (Fisher; model 505) at 30% amplitude, 1/1 s power cycling in an ice-water bath, 10 min per 6 l. Lysates were centrifuged at 17,540*g* for 45 min at 4 °C followed by syringe filtration (Millex; 0.45 μm).

Filtered lysates were loaded on Ni^2+^ columns (Cytiva HisTrap HP, 10 ml resin/6 l cell lysate) at 4 °C. The resin was then washed with four column volumes of loading buffer and equilibrated with cleavage buffer (15.1 mM Na_2_HPO_4_, 4.9 mM NaH_2_PO_4_, 20 mM imidazole, 100 mM NaCl, 2 mM tris(2-carboxyethyl)phosphine hydrochloride, 10% propylene glycol v/v, 300 μM phytic acid, pH 8.0) (57). Cleavage was allowed to occur overnight at 4 °C. The next day, cleaved protein was eluted with cleavage buffer. The protein fractions were pooled, and 1 ng of tobacco etch virus protease (60) was added. The sample was transferred to a 3.5 kDa molecular weight cutoff dialysis bag (Thermo Fisher Scientific) and dialyzed against 4 l of 20 mM MES (2-(4-morpholino)ethanesulfonic acid), 75 mM NaCl, 2.5 mM DTT, pH 6.0 for 3 h at room temperature.

Dialyzed samples were transferred to a conical vial, propylene glycol was added to 10% v/v, and the sample was centrifuged for 5 min at 4500*g*, 25 °C, to remove aggregates. The supernatant was processed using cation exchange chromatography at room temperature (Cytiva; SP High Performance). A custom 25 ml column was equilibrated with 25 mM MES, 4 M urea, 10% propylene glycol, and 120 mM NaCl. The column was then washed with 1 column volume of the equilibration buffer. A linear ramp to 300 mM NaCl over three column volumes was used to separate the Sos construct from closely related degradation products. Chromatography at 4 °C provides insufficient resolution; the full-length construct and degradation products interact and coelute. It is important to perform this step at room temperature.

SDS-PAGE with Coomassie visualization was used to identify sufficiently pure fractions. For storage, the selected fractions were combined and concentrated to about 1 mM and stored at −80 °C. The protein was exchanged into the appropriate buffer immediately before use. Determining the concentration of IPDs can be challenging (61); we estimated the molar absorptivity at 280 nm (15,470 [M cm]^−1^) (62). We further validated the identity and purity of the protein by high-resolution mass spectrometry (Supplemental Materials, Table S3).

### SH3

The native partner of Sos is the N-terminal SH3 domain of Drk (UniProt ID: Q08012). The wildtype sequence is partially unfolded under native conditions (63). To focus on binding alone, we used the stabilized T22G variant (64) labeled with a ^19^F atom on the single tryptophan (W36) in the Sos-binding site (29). Expression and purification were performed as described (24, 65), except that after size-exclusion chromatography, samples were polished *via* anion exchange chromatography to remove nucleic acids. A custom 25 or 50 ml Sepharose Q column (Cytiva) was equilibrated in 50 mM Tris (pH 7.4), the sample was loaded, and a linear ramp to 50 mM Tris, 450 mM NaCl was performed over two column volumes. Only SH3 eluted during using this protocol. DNA does not stick as well to the 5 ml prepacked columns from Cytiva and coelutes with SH3. Pure samples were extensively dialyzed into distilled and deionized water (>17 MΩ cm), aliquoted into amounts appropriate for each experiment, flash frozen in ethanol/CO_2_(s), and lyophilized for storage. Protein identity and purity were verified by mass spectrometry (Table S3).

### NMR

NMR data were acquired using Bruker Avance III HD spectrometers equipped with QCI cryoprobes (^1^H Larmor frequencies of 500, 470 MHz for ^19^F), or TCI cryoprobes (^1^H Larmor frequencies of 850 MHz, 213 MHz for ^13^C). Data were processed using NMRpipe (2020.171.18.39). Spectra across different temperatures are referenced to trimethylsilylpropanesulfonate.

^19^F spectra of Sos site 2 peptide (GenScript Biotech; >98% purity) were acquired using between 80 and 400 scans, with a 15 PPM sweep width, 1400 complex points, an interscan delay of 2.5 s, and a center frequency of −122.5 PPM. NMR samples were prepared as described (24). Titrations were performed three times: once using peptide concentrations of 0, 29, 73, 145, 218, 290, 435, 580, 870, 1160, and 1450 μM with an SH3 concentration of 290 μM, then twice with peptide concentrations of 0, 6, 16, 45, 64, 91, 128, 181, 256, 363, and 1450 μM and SH3 concentrations at 290 and 145 μM.

^1^H–^13^C correlation spectra were acquired using a heteronuclear multiple quantum correlation experiment (optimized for the methyl transverse relaxation optimized spectroscopy effect) (66) with between 8 and 40 scans, depending on the signal/noise of the Ile1384 peak. The ^1^H sweep width was 15 PPM using 5120 complex points and a center frequency at 4.7 PPM. Forty-eight complex points were collected in the indirect dimension, with a sweep width of 5 PPM, and a center frequency of 12.5 PPM. The water signal was suppressed and dephased using a combination of presaturation and selective shaped pulses. Sos site 4 protein was exchanged into 20 mM sodium phosphate, pH 7.5 plus 5% (v/v) D_2_O using a PD-10 midi desalting column (Cytiva); its concentration was then adjusted to 51.5 μM. Four hundred microliters of Sos site 4 solution were used to dissolve lyophilized SH3 for the initial titration point, and then spectra were acquired at 4.2, 15, 25, 35, and 45 °C. The solution containing only Sos site 4 was used for a series of dilutions to obtain SH3 titration points of 270, 122, 100, 75.0, 50.0, 37.5, 25.0, 12.5, 6.25, 3.13, 1.56, 0.781, 0.391, and 0 μM.

### NMR titration data analysis

1D NMR titration measurements of peptides with ^19^F-labeled SH3 were fit to a two-state interaction model (peptide+SH3⇌complex) using ^19^F lineshape analysis, as described (24). The uncertainties in dissociation constants and dissociation rates are the standard error of triplicate measurements. Measurements across multiple temperatures were then fitted to van’t Hoff and Eyring equations to determine *Δ*$H_{D}^{{}^{\circ}\prime }$, T*Δ*$S_{D,298.15K}^{{}^{\circ}\prime }$, *Δ*$H_{A}^{{}^{\circ}\prime ‡}$, T*Δ*$S_{A,298.15\;K}^{{}^{\circ}\prime ‡}$, *Δ*$H_{D}^{{}^{\circ}\prime ‡}$, and T*Δ*$S_{D,298.15K}^{{}^{\circ}\prime ‡}$, with uncertainties determined by the standard deviations of bootstrap analysis with 1000 replicas (MATLAB 2021b).

^1^H–^13^C heteronuclear multiple quantum correlation titration measurements of SH3 into [^13^CH_3_-Ile]-labeled Sos were analyzed using NMR TITAN, version 1.6 (34). Measurements for each temperature were fit to a two-state interaction model, and parameter uncertainties were estimated from 100 bootstrapped replicas (34, 67). Ile1384, Ile1394, Ile1325, and one pseudo peak were fit as a single spin group to account for overlapping resonances. Measurements across multiple temperatures were then fitted to the van’t Hoff and Eyring equations to determine *Δ*$H_{D}^{{}^{\circ}\prime }$, T*Δ*$S_{D,298.15K}^{{}^{\circ}\prime }$, *Δ*$H_{A}^{{}^{\circ}\prime ‡}$, T*Δ*$S_{A,298.15\;K}^{{}^{\circ}\prime ‡}$, *Δ*$H_{D}^{{}^{\circ}\prime ‡}$, and T*Δ*$S_{D,298.15K}^{{}^{\circ}\prime ‡}$. Errors generated from TITAN fits were used to perform weighted least squares regressions, and the reported uncertainties are the 95% confidence intervals of fits.

### ITC

Each set of protein samples were exchanged using PD-10 midi desalting columns and then dialyzed overnight into the same solution of 20 mM sodium phosphate, pH 7.5 using dialysis cassettes (Thermo Fisher Scientific; 2 kDa molecular weight cutoff). Samples were filtered (Millex; 0.22 μm) prior to measurement, and concentrations were validated by measuring the absorbance at 280 nm (Thermo Fisher Scientific; NanoDrop One).

ITC was performed using a MicroCal PEAQ-ITC Automated (Malvern Panalytical), with Sos in the cell and SH3 as the ligand, with one 0.4 μl injection, followed by 19, 2 μl injections. At 4.2 °C, the cell concentration was 229 μM, and the ligand concentration was 4.00 mM for site 2. For site 4, the cell concentration was 250 μM, and the ligand concentration was 3.56 mM. At 35 °C, the cell concentration was 229 μM, and the ligand concentration was 4.00 mM for site 2. For site 4, the cell concentration was 350 μM, and the ligand concentration was 4.67 mM. Knockout measurements were concentration matched and made alongside each measurement described.

### ITC data analysis

Data were analyzed with PEAQ-ITC Analysis Software (Malvern Panalytical). To distinguish specific binding from nonspecific binding, knockout replicates were used as controls and subtracted from the corresponding Sos replicate. Measurements for each construct were fit to a single-site model.

## Data availability

All data are contained within the article.

## Supporting information

This article contains supporting information (24, 30, 68, 69).

## Conflict of interest

The authors declare no conflicts of interests with the contents of this article.
