# Supplementary material for: Disordered proteins mitigate the temperature dependence of site-specific binding free energies
Source: J Biol Chem. 2023 Feb 3;299(3):102984. doi: 10.1016/j.jbc.2023.102984 (PMC10027511; doi:10.1016/j.jbc.2023.102984)
Supplement: Supporting information [file mmc1.pdf]

Supporting information

Disordered proteins mitigate the temperature dependence of site-specific binding free energies

Joseph F. Thole, Christopher A. Waudby and Gary J. Pielak*

For correspondence: Gary J. Pielak, gary_pileak@unc.edu

^{13}C-δ1methyl peak assignments

Sos of sevenless Site 4 variants I1325N and I1394L were designed to determine cross-peak identity in the ^{1}H-^{13}C TROSY-HMQC measurements. Template DNA purchased from Gene Universal (Newark, DE) was transformed into DH5α competent cells (Thermo Fisher Scientific, Waltham, MA). A 25 mL culture were grown overnight at 37 °C in Lennox broth and 50 mg/L kanamycin (LB, 10 g/L tryptone, 5 g/L yeast extract, 5 g/L NaCl). The next day, plasmid DNA was harvested using a QIAprep Spin Miniprep kit (Qiagen, Hilden, Germany), following the manufacturer's protocol.

To generate the I1325N variant, the forward primer GTGCGGTGCCGAACAGCC and reverse primer CGTGCGGGCTGTTCGGC were added at 500 nM to a polymerase chain reaction (PCR) using Q5 2X master mix (New England Biolabs, Ipswitch MA),1 ng/uL Site 4 purified template and 2% dimethyl sulfoxide (DMSO) v/v. I1394L was generated using the same recipe, but with forward primer GAACCACAGCACCGGCCTTAGCTAT, reverse primer GACGCAGATAGCTAAGGCCGGT, and with 6% DMSO. PCR carried out following the manufacturer's protocol using a 72°C annealing temperature.

After PCR, samples were treated with 10 U DpnI (Thermo Fisher Scientific) for 1 hour at 37 °C, followed by inactivation at 80 °C for 20 min. PCR success was followed-up with sanger sequencing (GENEWIZ, South Plainfield, NJ) of the plasmid region encoding the entire protein product to ensure the correct mutation and no other changes.

The mutant DNA was then transformed into BL21 cells (Thermo Fisher Scientific) and 1 L of each was expressed, purified and prepared for NMR as described in the main text. The I1325N and I1394L variants were measured at 29 and 5 μM. Peaks were referenced to I1384 (present in both variants) and the missing peak was identified as the variant residue (Figure S4).

^{19}F NMR

Data were acquired using Bruker Avance III HD spectrometers equipped with a QCI cryoprobe (^{1}H Larmor frequencies of 500, and 470 MHz for ^{19}F). Data was processed using NMRpipe (2020.171.18.39) and referenced to trimethylsilylpropanesulfonate to account the temperature dependence of chemical shift.

^{19}F spectra comprised at least 80 scans or until the signal/noise ratio reached 10 (with a line broadening of 2 Hz), a 15 PPM sweep-width, 1400 points and an interscan delay of 2.5 s with a center frequency of -122.5 PPM. Sos site 4 (non-MBD tagged construct) was exchanged into of 50 mM HEPES, 50 mM bis-tris propane, 50 mM sodium acetate pH 7.5 plus 5% (v/v) D_2O using a PD-10 Midi column (Cytiva) selecting the fractions with the highest concentration for the titration. The Sos solution was used to dissolved one aliquot of SH3, and a second, larger

volume solution of SH3 was prepared in the same buffer, at an identical concentration of SH3. Measurements were made at 4.2, 15, 25, 35 and 45 °C. The sample was then removed, diluted using the larger SH3 solution and returned to a clean tube. Site 4 titration concentrations were 0, 0.29, 2.9, 7.3, 15, 29, 73, 145, 218, 290, 435, 870 and 1160 μM, with SH3 at 270 μM (Figure S3, Table S2).

We also attempted to fit titration data to a bi-dentate binding model, describing binding of SH3 to two sites on Sos, using numerical solutions of Bloch-McConnell equations in Julia as previously described (69) and originally implemented in NMR TITAN (70). In this model, SH3 bound to two sites on the Sos titrant, without cooperativity, described by two dissociation constants and dissociation rates. Chemical shifts within bound states were independent of binding to the second site, but linewidths were allowed to vary, reflecting differences in molecular weight of the complex. Data were fitted globally across temperatures, following van't Hoff and Eyring equations and assuming linear changes in chemical shifts with temperature. Reported uncertainties are derived from the curvature of the χ^2 surface, although because the model did not fully describe the observed results, as discussed in the main text, these should be regarded with caution.

Buffer and construct comparisons

The Site 2 peptide was measured in 50 mM HEPES, 50 mM bis-tris propane, 50 mM sodium acetate, pH 7.5, as well as the previous work on the Drosophila melanogaster Sos peptides.(71,72) To determine how buffer conditions might affect the equilibrium, we performed ITC at 4.2 °C with non-MBP tagged Sos as described in the main text, with Sos Sites 2, 4 and knockout at 250 μM, and SH3 at 3.75 mM (Table S2). The results show a similar decrease, but smaller in magnitude decrease in enthalpy relative to 20 mM sodium phosphate, with a similar affinity to that of the peptides.

To determine if an additional serine on the N-terminus of the Sos construct (MBP-tagged Sos results in an extra SMG at the N-term, while the non-MBP tagged construct has an extra MG), we performed ITC 4.2 °C in 20 mM sodium phosphate, pH 7.5, with Sos-SMG, Sos-MG, and knockout controls. Experimental parameters are described in Table S2.

Determining effects of alanine knockouts

ITC measurements of Wildtype Sos (sites 1-4) and sites 2 and 4 (sites 1 and 3 knocked out) were made in in 50 mM HEPES, 50 mM bis-tris propane, 50 mM sodium acetate, pH 7.5 with cell concentrations of 225 μM and SH3 syringe concentrations of 7.17 mM SH3. One 0.4 μL injection was made, following by thirty-eight 1 μL injections.

To simplify analysis, after knockout subtraction, the sites 2 and 4 construct was fit to a single site (Figure S6B), given the similarities of individual site 2 and site 4 constructs (Figure S6D and S6E). The stoichiometry of 2.6 is likely due to small errors in quantifying the initial Sos protein concentration that is amplified by having two binding sites on each molecule.

The presence of multiple binding sites with varied affinities made the quantification of Sos sites 1-4 (Figure S6A) challenging, however the consistency of sites 2 and 4 individually, and together led us to fit the binding to a two-site model after knockout subtraction, with one site constrained by the affinity of sites 2 and 4, and the second site allowed to float. From this approach, we see that the constrained, stronger sites (2 and 4) have a stoichiometry of 1.6 ± 0.2 and thermodynamic parameters that are in line with the fits of the other constructs (Table S2). The second fitted site has a stoichiometry of 1.1 ± 0.2, with a K_D of 24 ± 5 μM, a $\Delta H_D^{\circ\prime}$ of 6.0 ± 0.7 kcal/mol and a $T\Delta S_D^{\circ\prime}$ of -0.1 ± 0.4 kcal/mol (Table S2). Based on our previous peptide study results, site 3 is the most likely candidate to explain this binding event (72), as site 1 had mM affinity even at 5 °C. These results suggest that the alanine substitutions effectively knockout specific binding at the targeted sites and that we are measuring binding at the specific sites of interest.

Mass spectrometry analysis

All mass spectrometry measurements were performed on a Thermo QExactive HF equipped with an electrospray ionization source. Purified protein was diluted to 8 μM with 2.5 ammonium acetate. The proteins were directly infused using a syringe pump at 3 uL/min and spray voltage was set to 3.2 kV. MS scans were collected across a scan range from 1000 to 4000 m/z. Scans were averaged over 3 microscans and collected at 30K resolution. Mass spectra were deconvolved using Protein Metrics Byos software.

Table S1. Comparison of ITC results by Sos construct and buffer.

Sos construct		Temperature (°C)	Buffer	[Sos] (mM)	[SH3] (mM)	N	K_D (μM) [1]	$\Delta H^{o'}_D$ (kcal/mol) [1]	$\Delta G^{o'}_D$ (kcal/mol) [2]	$T\Delta S^{o'}_D$ (kcal/mol) [2]	Offset[1]	Sign of $\Delta C_{P,D}$
2	MG	4.2	HBA[3]	0.250	3.75	1.08 ± 0.01	9 ± 1	4.96 ± 0.08	6.43 ± 0.05	-1.5 ± 0.2	-0.46 ± 0.03	positive
	MG		NaPi[4]	0.229	4.00	1.23 ± 0.03	7 ± 2	3.2 ± 0.2	6.56 ± 0.03	-3.47 ± 0.07	-0.53 ± 0.06	
	SMG		NaPi[4]	0.229	4.00	1.24 ± 0.03	5 ± 2	2.5 ± 0.1	6.76 ± 0.03	-4.25 ± 0.07	-0.26 ± 0.06	
	SMG	35.1	NaPi[4]	0.229	4.00	1.21 ± 0.01	14 ± 1	8.9 ± 0.1	6.86 ± 0.01	2.1 ± 0.1	0.00 ± 0.05	
4	MG	4.2	HBA[3]	0.250	3.75	1.10 ± 0.03	8 ± 4	4.7 ± 0.3	6.5 ± 0.3	-1.8 ± 0.8	-0.4 ± 0.1	positive
	SMG		NaPi[4]	0.250	3.56	1.23 ± 0.02	3 ± 1	2.9 ± 0.1	6.95 ± 0.02	-4.10 ± 0.03	-0.47 ± 0.07	
	SMG	35.1	NaPi[4]	0.350	4.67	1.29 ± 0.02	14 ± 3	8.4 ± 0.3	6.84 ± 0.02	1.5 ± 0.1	-0.5 ± 0.2	
2 and 4	SMG	4.2	HBA[3]	0.225	7.17	2.64 ± 0.03	7 ± 2	4.7 ± 0.1	6.50 ± 0.01	-1.83 ± 0.03	-0.22 ± 0.06	
WT[3]	SMG	4.2	HBA[3]	0.225	7.17	1.6 ± 0.2	8 ± 3	4.1 ± 0.5	6.48 ± 0.04	-2.4 ± 0.3	-0.14 ± 0.03	
						1.1 ± 0.2	24 ± 5	6.0 ± 0.7	5.9 ± 0.2	-0.1 ± 0.4		
Knockout	SMG	4.2	HBA[3]	0.225	7.17	1 ± 2	1800 ± 700	10 ± 20	3.5 ± 0.1	11 ± 7	0.04 ± 0.07	

[1]Uncertanties derived from error propagation of three fits. [2]Uncertainties are the standard deviation of three estimates. [3]50 mM HEPES, 50 mM bis-tris propane, 50 mM sodium acetate, pH 7.5. [4]20 mM sodium phosphate, pH 7.5. [3]Wildtype Sos fit was constrained using the affinities derived from site 2, site 4 and sites 2 and 4.

Table S2. Fit parameters of ^{19}F SH3 titration fits of bidentate fits.

	Site 1					Site 2					Free State	
T (°C)	K_D (μM)	k_{off} (10^3 s^{-1})	k_{on} (10^8 s^{-1} M^{-1})	δ (ppm)	R_2 (s^{-1})	K_D (μM)	k_{off} (10^3 s^{-1})	k_{on} (10^8 s^{-1} M^{-1})	δ (ppm)	R_2 (s^{-1})	δ (ppm)	R_2 (s^{-1})
4.2	2.15 ± 0.04	0.080 ± 0.002	0.37 ± 0.01	-123.2440 ± 0.0006	290 ± 2	800 ± 200	11 ± 4	0.15 ± 0.07	-122.5 ± 0.4	1000 ± 300	-123.863 ± 9e-5	115.4 ± 0.6
15	4.90 ± 0.08	0.239 ± 0.003	0.49 ± 0.01	-123.2330 ± 0.0005	191 ± 1	1500 ± 300	15 ± 4	0.10 ± 0.03	-122.3 ± 0.3	600 ± 200	-123.863 ± 6e-5	90.6 ± 0.3
25	10.6 ± 0.2	0.667 ± 0.007	0.63 ± 0.01	-123.2210 ± 0.0005	136 ± 1	3000 ± 500	19 ± 5	0.06 ± 0.02	-122.0 ± 0.3	0 ± 300	-123.863 ± 5e-5	75.3 ± 0.4
35	21.7 ± 0.3	1.74 ± 0.02	0.80 ± 0.02	-123.2100 ± 0.0005	106 ± 2	6000 ± 1000	23 ± 7	0.04 ± 0.01	-121.8 ± 0.3	200 ± 400	-123.863 ± 6e-5	65.2 ± 0.3
45	42.6 ± 0.8	4.29 ± 0.09	1.01 ± 0.03	-123.1990 ± 0.0006	94 ± 2	10000 ± 2000	30 ± 10	0.03 ± 0.01	-121.5 ± 0.4	0 ± 700	-123.863 ± 9e-5	64.3 ± 0.4

Table S3. Theoretical and measured masses of proteins.

Protein	Theoretical mass (Da)	Average mass observed (Da)	Mass error (PPM)
SH3	6833.56	6832.9	-96.58
Sos site 2	24703.24	24702.9	-13.76
Sos site 4	24618.14	24617.4	-30.06
Sos knockout	24490.95	24490.5	-18.37

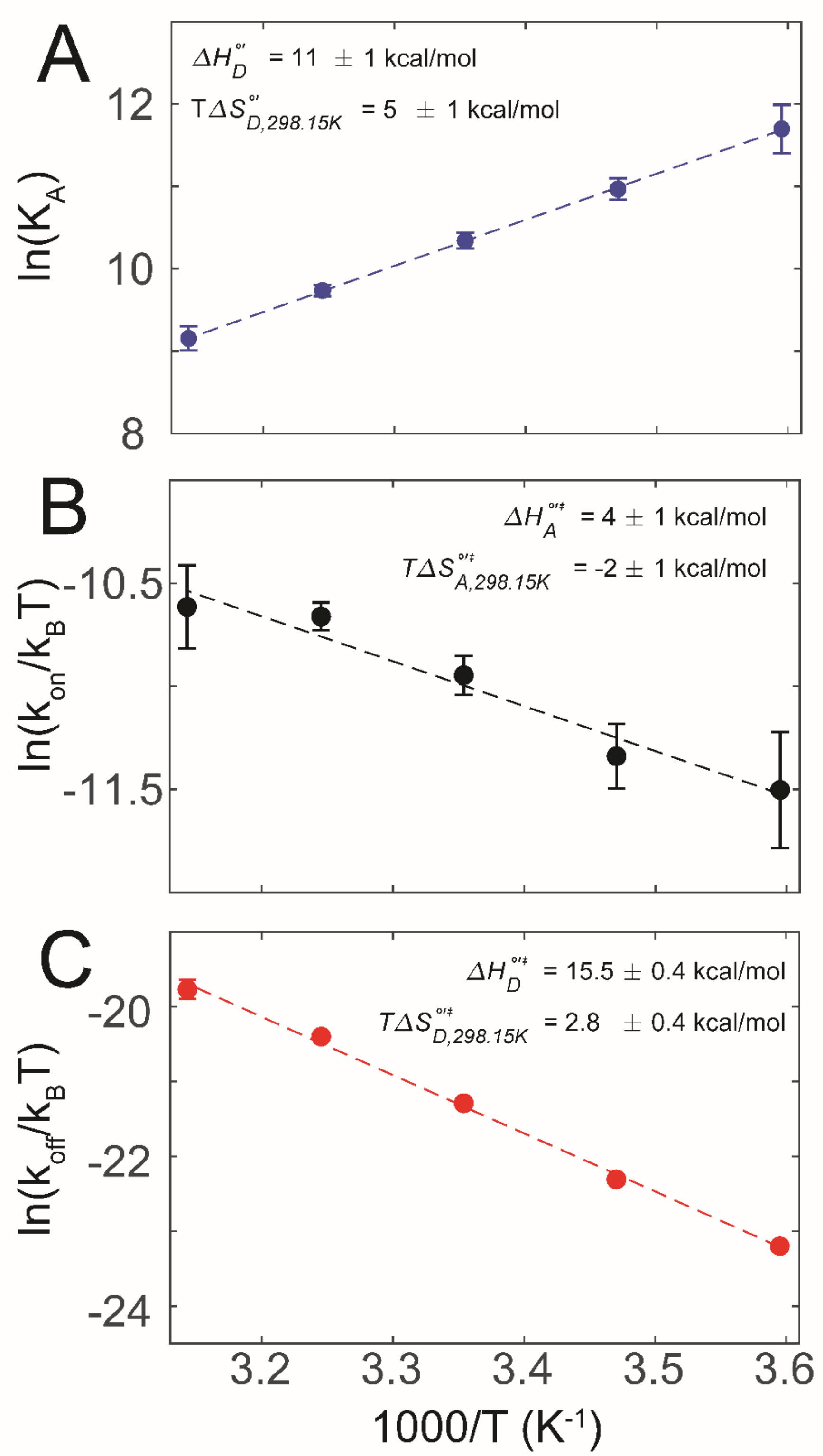

Figure S1. Analysis of SH3 - Sos site 2 peptide binding. A) van't Hoff analysis B) Eyring plots of association C) and dissociation. Error bars are the SEM of triplicate measures. Values for enthalpy and entropy are the mean and standard deviation of bootstrap analysis. Dotted lines show fits of the bootstrapped parameters. Measurements were made at a constant concentration of 290 or 145 µM SH3, and Sos site 2 peptide concentrations of 0, 29, 73, 145, 218, 290, 435, 580, 870, 1160, and 1450 µM or 0, 6, 16, 45, 64, 91, 128, 181, 256, 363, and 1450 µM.

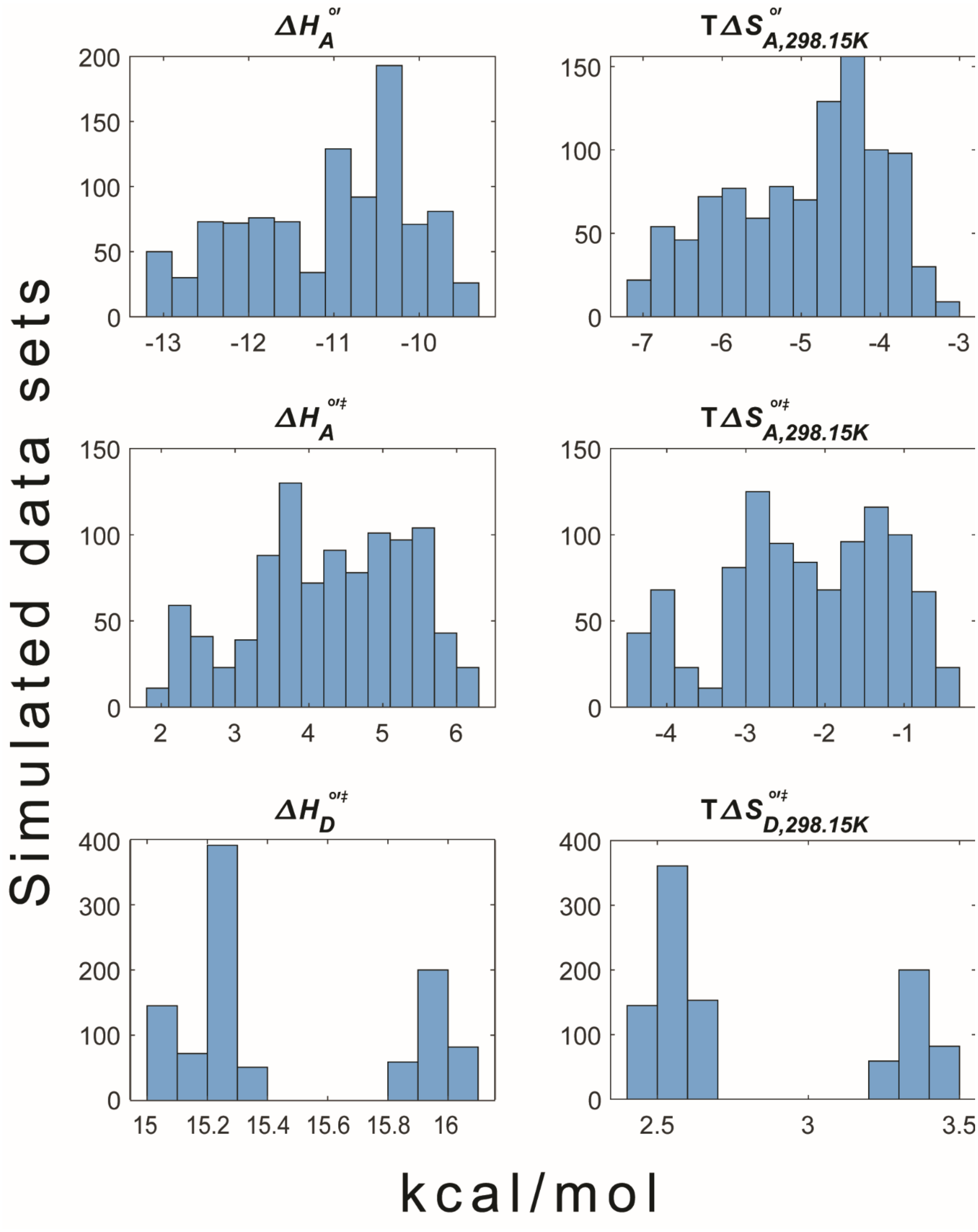

Figure S2. Distributions of parameters for 1000 bootstrapped fits to the A) Van't Hoff equation B) Erying equation for association and C) Eyring equation for dissociation of SH3-Sos site 2 peptide binding.

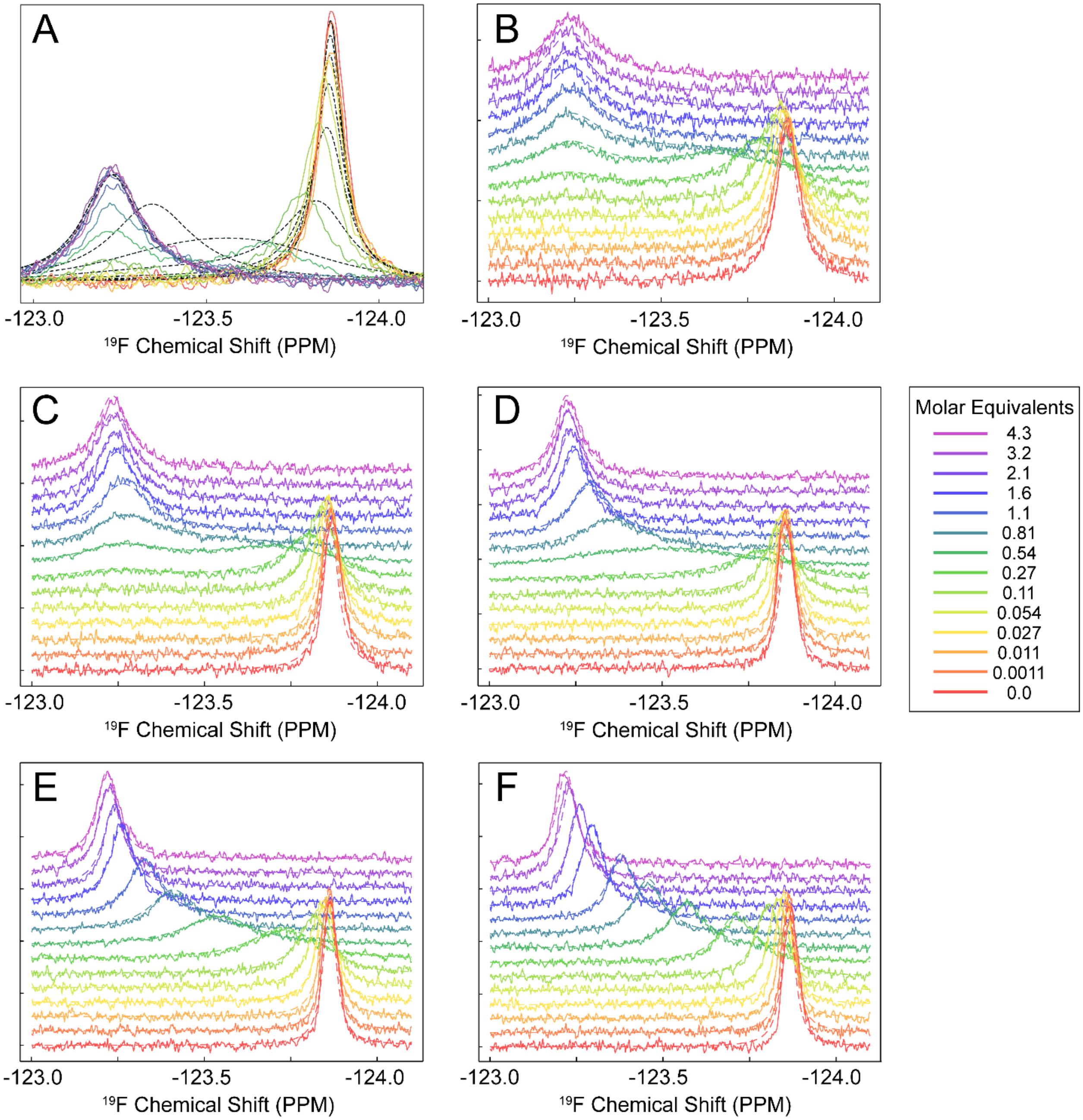

Figure S3. Titration of Sos site 4 into SH3. Titration was performed at A,B) 4.2 °C, C) 15 °C, D) 25 °C, E) 35 °C, F) 45 °C. Sos site 4 concentration was 270 µM, and SH3 concentrations were 0, 0.29, 2.9, 7.3, 15, 29, 73, 145, 218, 290, 435, 870 and 1160 µM. Panel A shows a fit to a two-state model (K_D = 0.3 µM, k_{on} = 2.3*10^9 $(s \cdot M)^{-1}$ k_{off} = 6.91*10^2 s^{-1}), B-F shows fits to a bidentate model. Solid lines are the real spectra with fitted spectra represented by dashed lines.

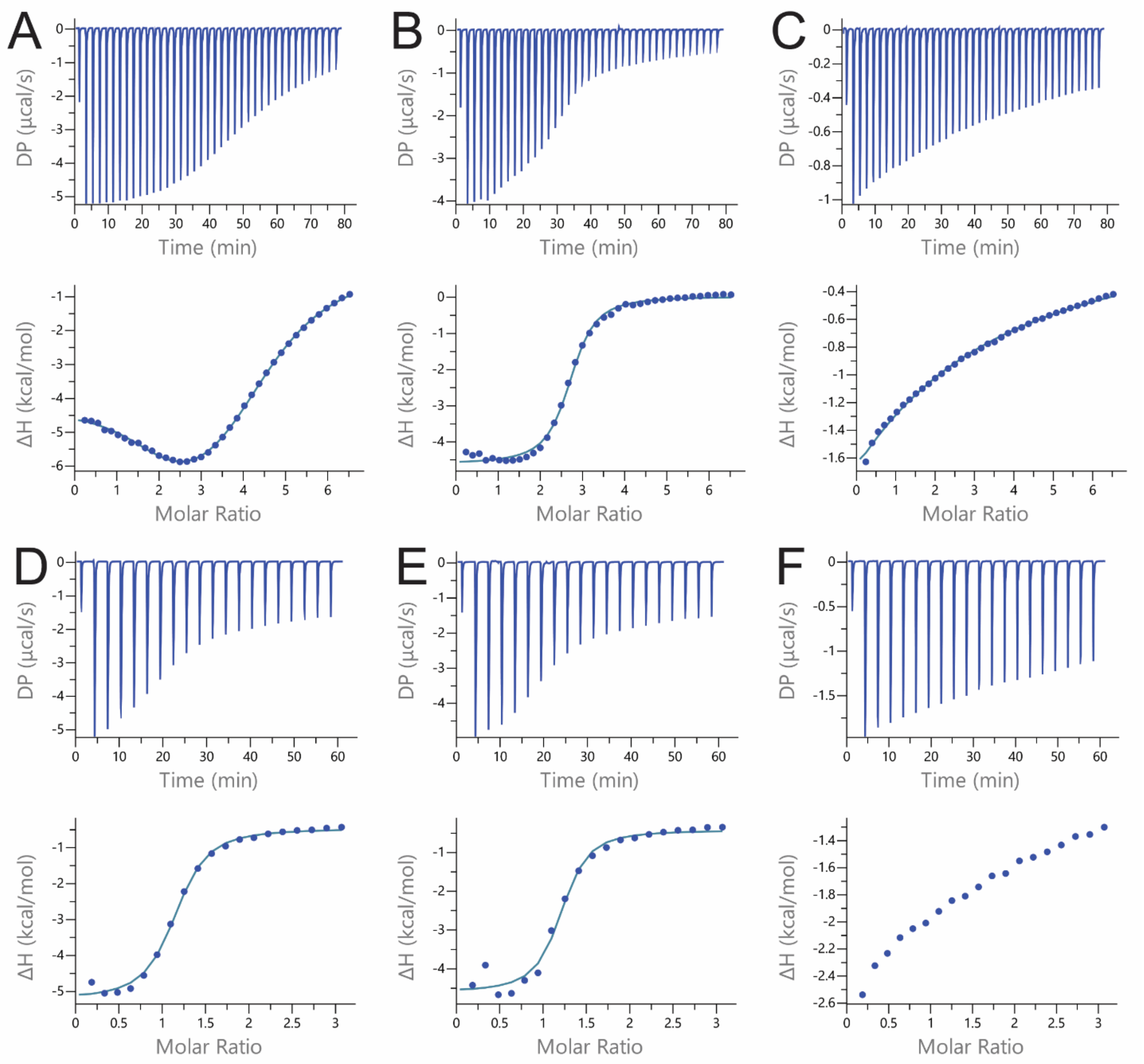

Figure S4. ITC thermograms of A) Wildtype Sos, B) Sos sites 2 and 4, C) matched knockout of A and B, D) Sos site 2, E) Sos site 4, F) matched knockout of D and E. All measurements were made at 4.2 °C in 50 mM HEPES, 50 mM bis-tris propane, 50 mM sodium acetate, pH 7.5. A, B and C had cell concentrations of 225 µM and SH3 syringe concentrations of 7.17 mM. D, E, and F had cell concentrations of 250 µM and SH3 syringe concentrations of 3.75 mM. A-E are quantified in Table S2.

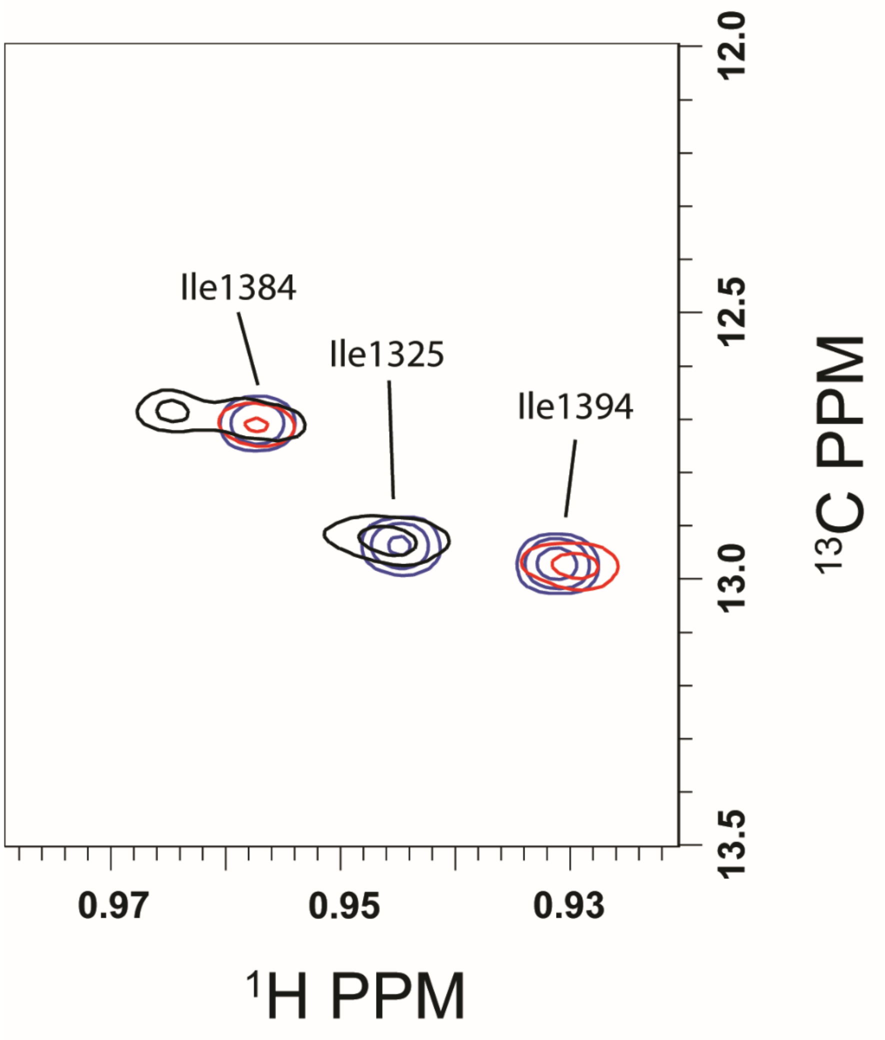

Figure S5. Overlay of 13C-δ1methyl isoleucine spectra of Sos site 4 (Blue), site 4 I1325N (Red) site 4 I1394L (Black) at 45

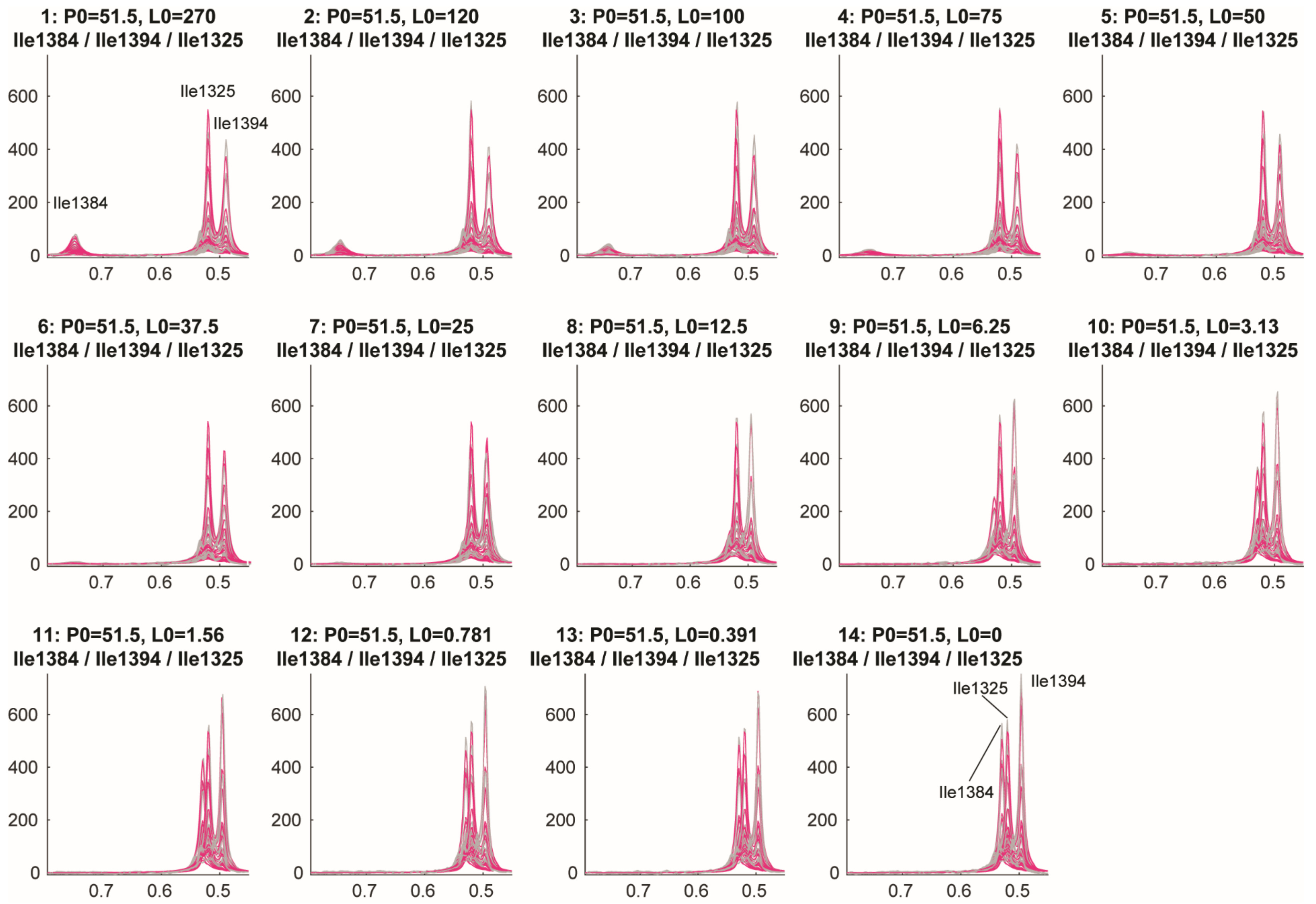

Figure S6. Overlay of real (grey) and simulated (pink) spectra from lineshape analysis of 5 °C ^{1}H-^{13}C titration of Sos site 4. X-axis is ^{1}H chemical shift in PPM. P0 is the concentration of Sos site 4 and L0 is the concentration of SH3 in μM.

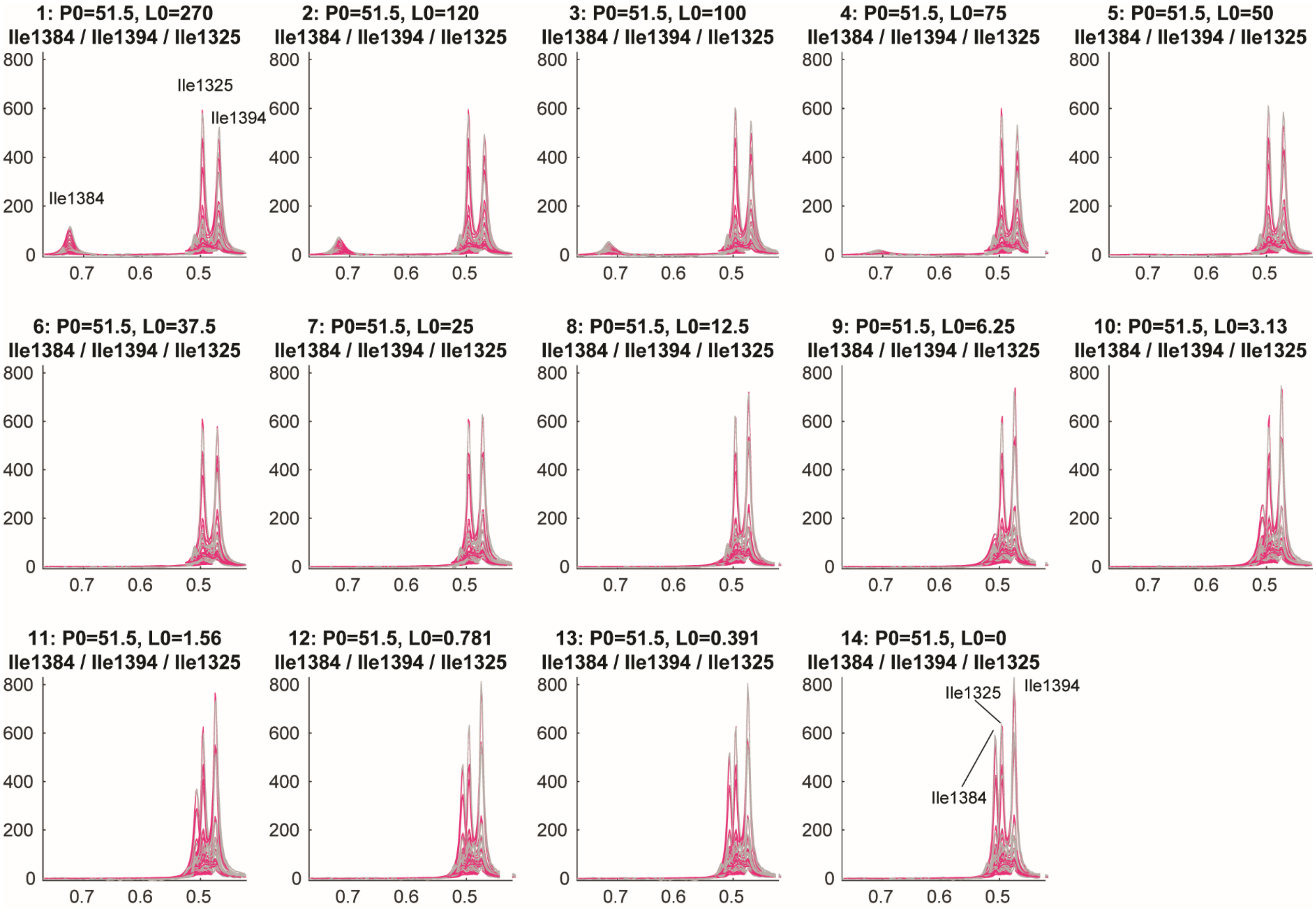

Figure S7. Overlay of real (grey) and simulated (pink) spectra from lineshape analysis of 15 °C ^{1}H-^{13}C titration of Sos site 4. X-axis is ^{1}H chemical shift in PPM. P0 is the concentration of Sos site 4 and L0 is the concentration of SH3 in μM.

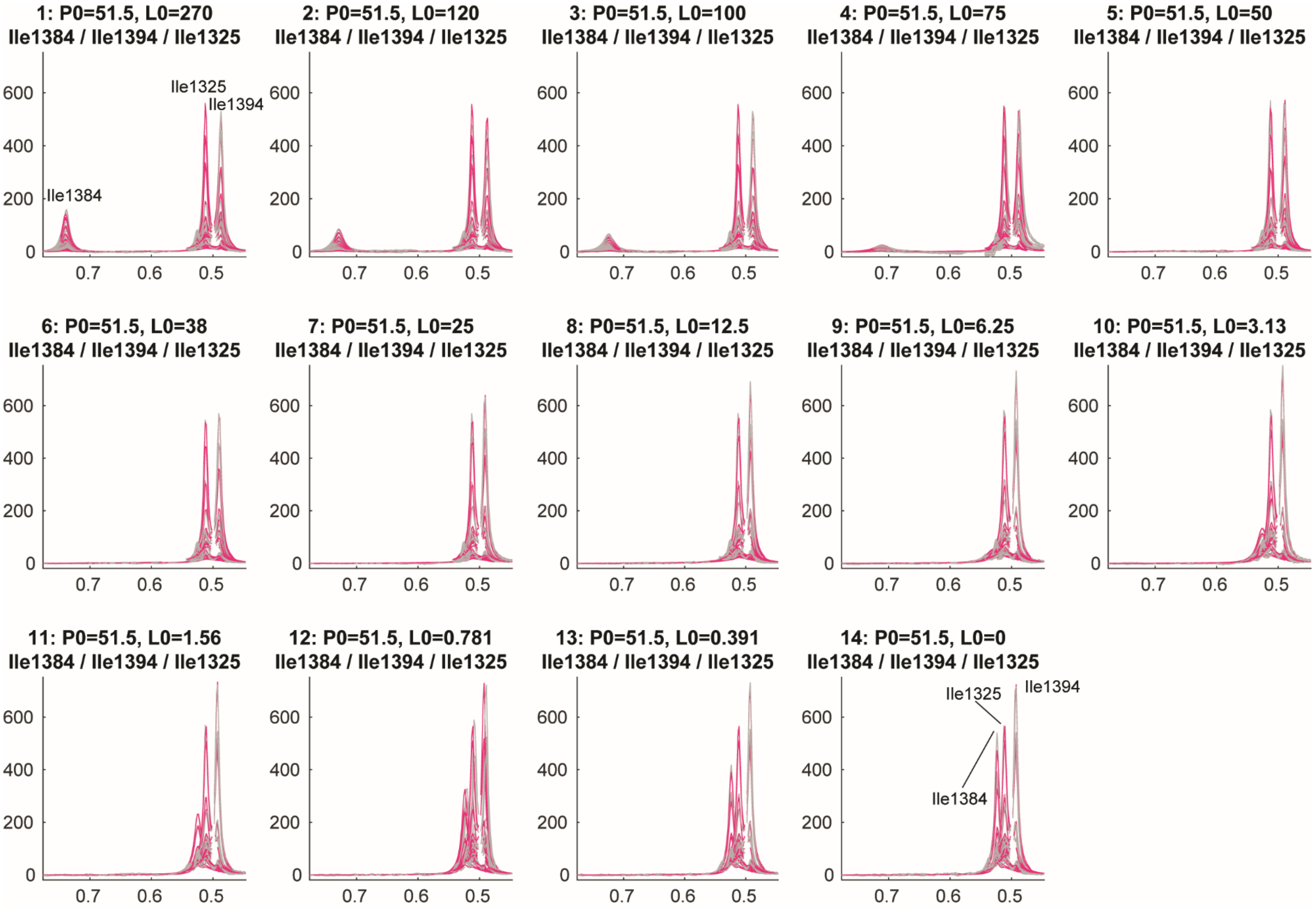

Figure S8. Overlay of real (grey) and simulated (pink) spectra from lineshape analysis of 25 °C ^{1}H-^{13}C titration of Sos site 4. X-axis is ^{1}H chemical shift in PPM. P0 is the concentration of Sos site 4 and L0 is the concentration of SH3 in μM.

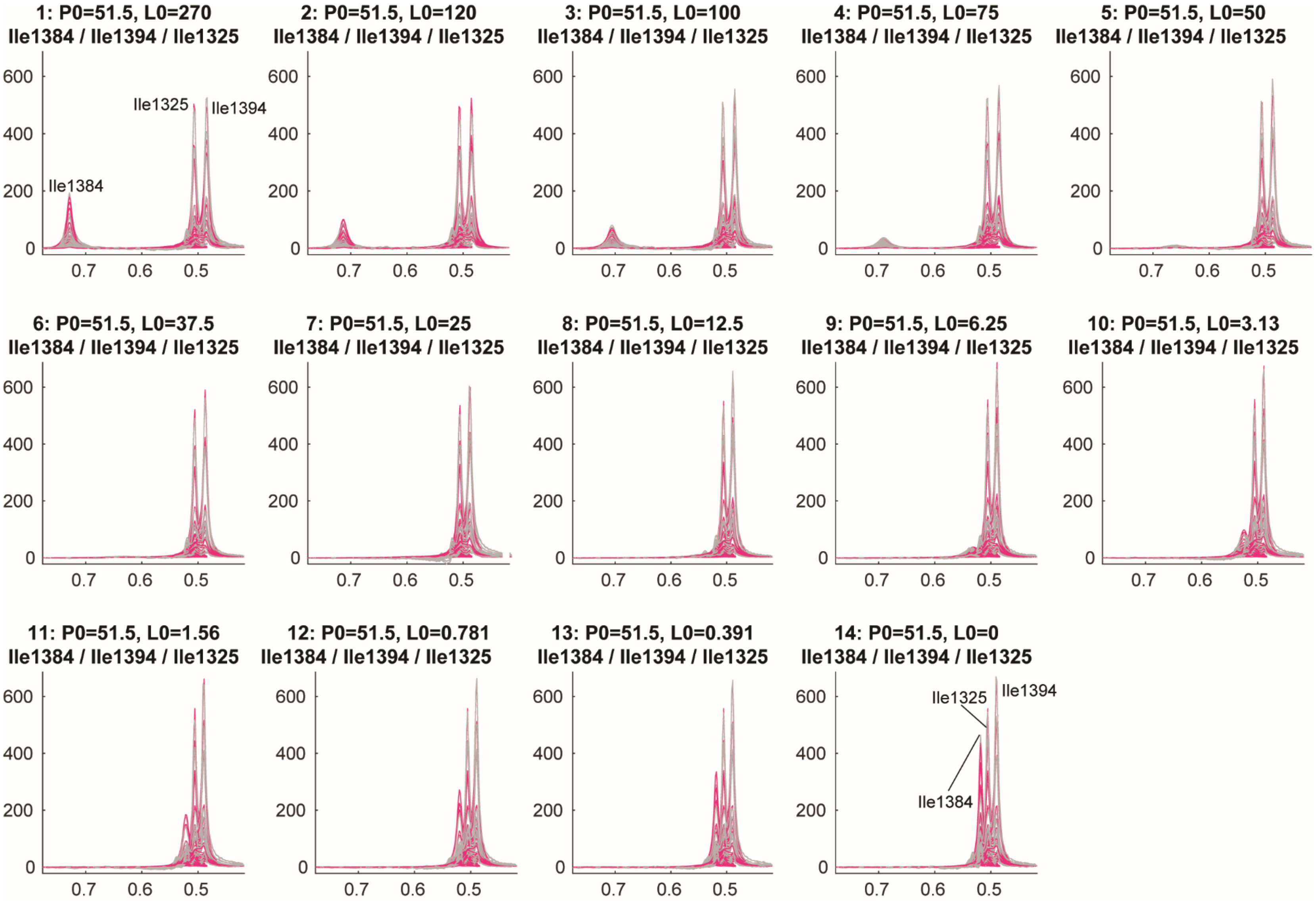

Figure S9. Overlay of real (grey) and simulated (pink) spectra from lineshape analysis of 35 °C ^{1}H-^{13}C titration of Sos site 4. X-axis is ^{1}H chemical shift in PPM. P0 is the concentration of Sos site 4 and L0 is the concentration of SH3 in μM.

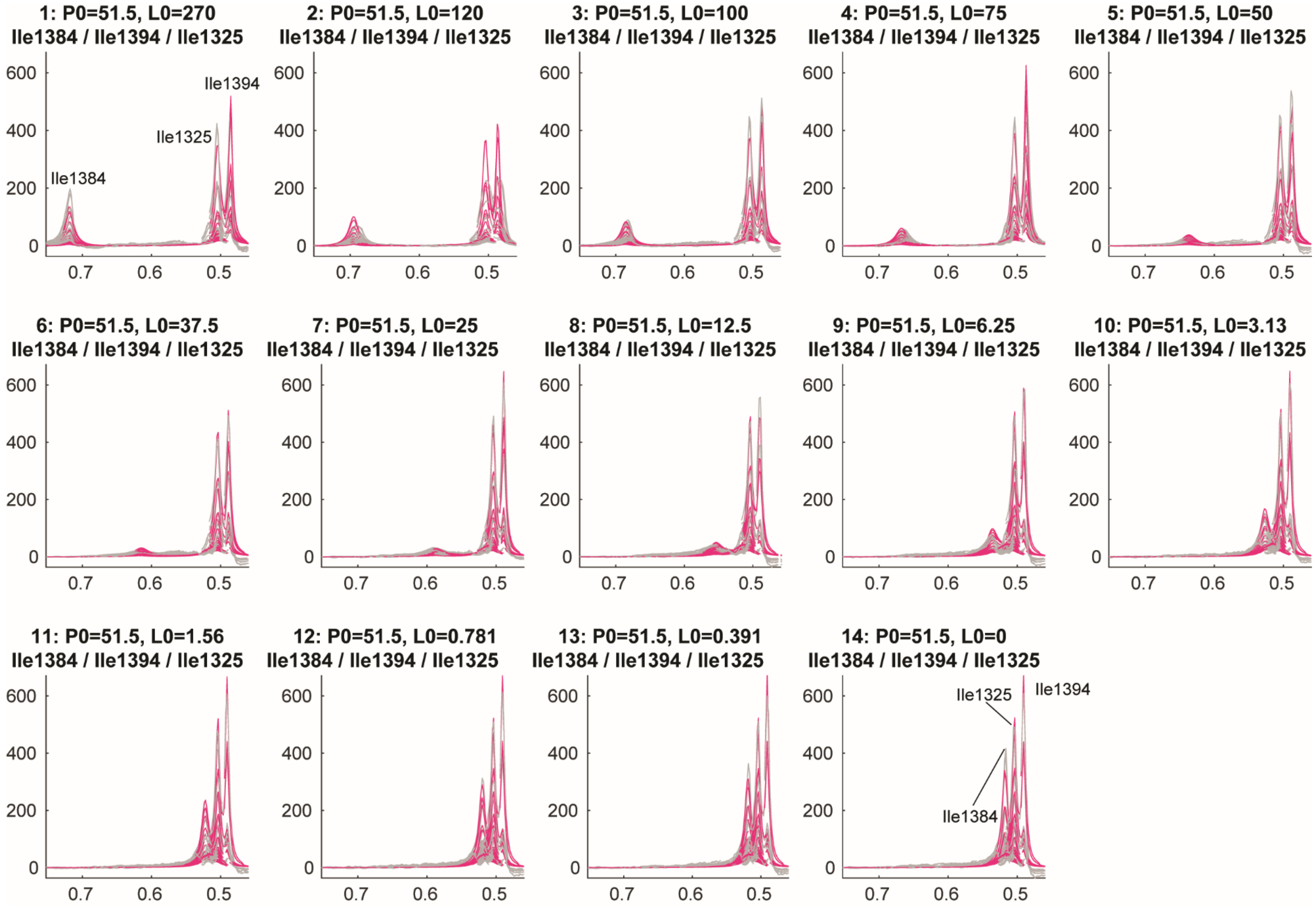

Figure S10. Overlay of real (grey) and simulated (pink) spectra from lineshape analysis of 5 °C ^{1}H-^{13}C titration of Sos site 4. X-axis is ^{1}H chemical shift in PPM. P0 is the concentration of Sos site 4 and L0 is the concentration of SH3 in μM.

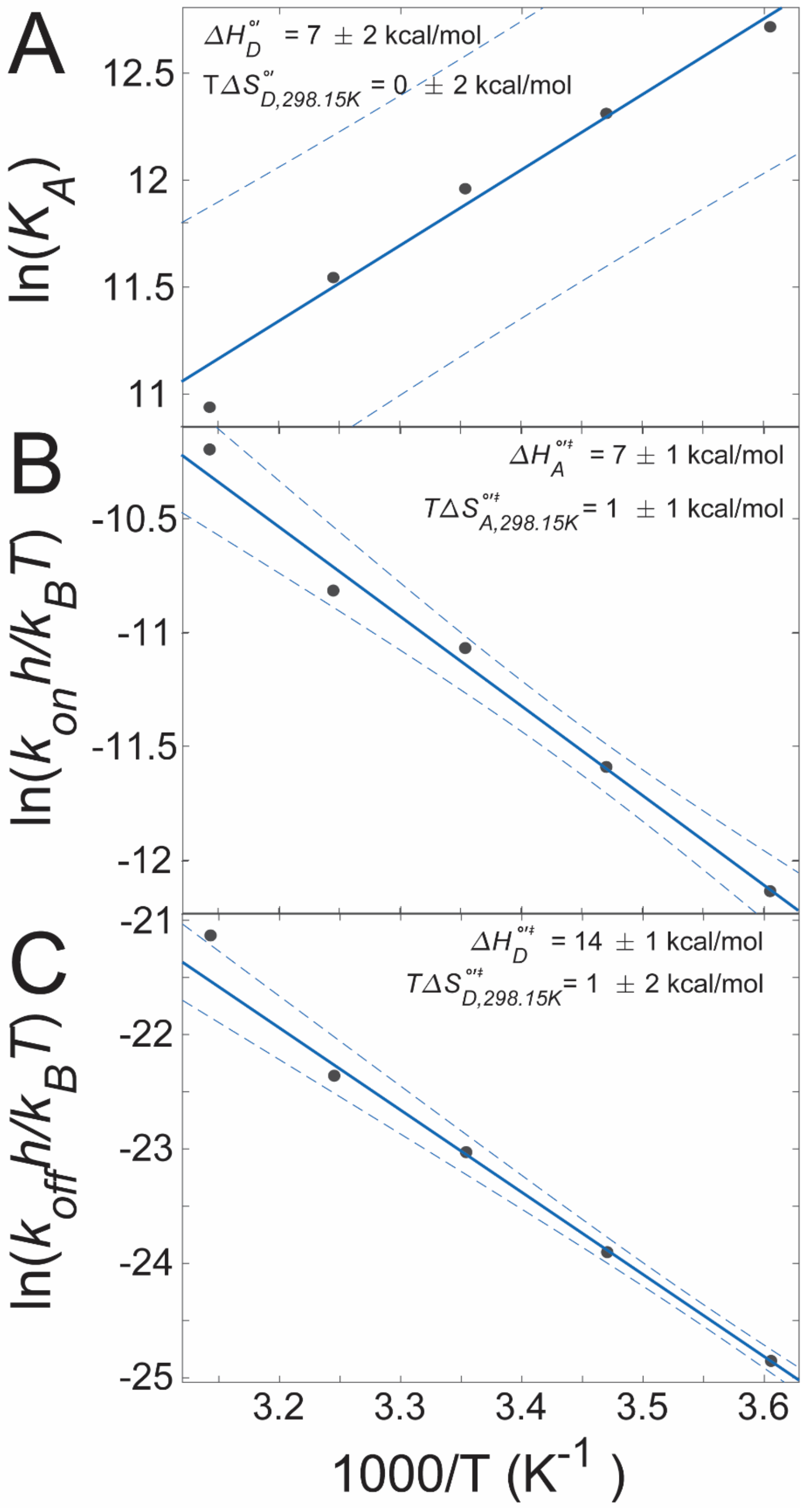

Figure S11. Fits of ^{1}H-^{13}C δ1methyl isoleucine lineshape analysis parameters. A) van't Hoff analysis B) Eyring plots of association C) and dissociation. Dotted lines indicated 95% Confidence intervals of the fit, with uncertainties derived from the 95% confidence intervals of each fit parameter

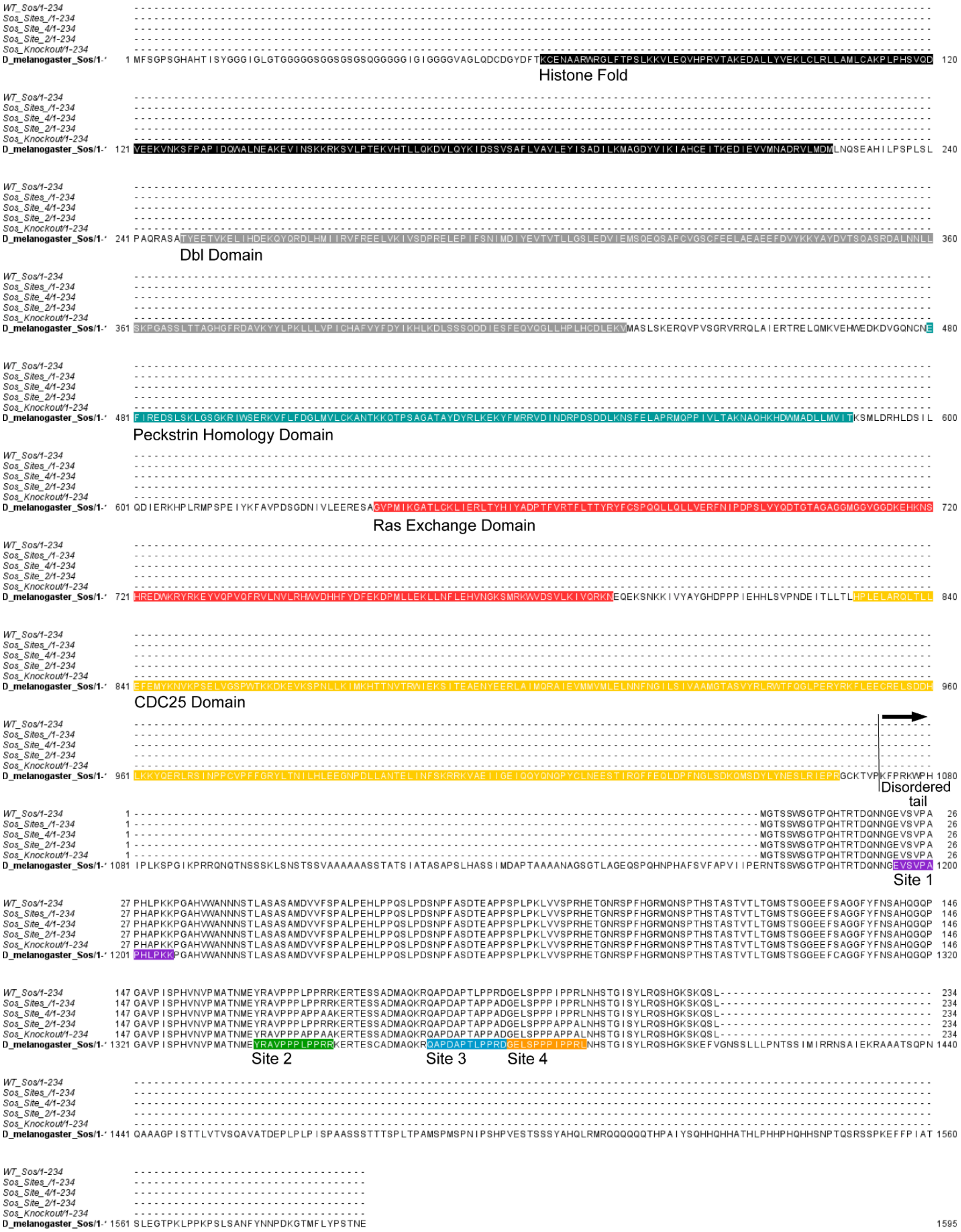

Figure S12. Sequence alignment WT Sos, Sos site 2, Sos site 4, Sos Sites 2 and 4, Sos knockout and the D. melanogaster Son of sevenless. Sequence alignment performed in Jalview v2.11

References

69. Pavlović, R. Z., Lalisse, R. F., Hansen, A. L., Waudby, C. A., Lei, Z., Güney, M., Wang, X., Hadad, C. M., and Badjić, J. D. (2021) From selection to instruction and back: Competing conformational selection and induced fit pathways in abiotic hosts. *Angew. Chem., Int. Ed.* **60**, 19942-19948

70. Waudby, C. A., Ramos, A., Cabrita, L. D., and Christodoulou, J. (2016) Two-dimensional NMR lineshape analysis. *Sci. Rep.* **6**, 24826

71. Stadmiller, S. S., Aguilar, J. S., Parnham, S., and Pielak, G. J. (2020) Protein-peptide binding energetics under crowded conditions. *J. Phys. Chem. B* **124**, 9297-9309

72. Stadmiller, S. S., Aguilar, J. S., Waudby, C. A., and Pielak, G. J. (2020) Rapid quantification of protein-ligand binding via ^{19}F NMR lineshape analysis. *Biophys. J.* **118**, 2537-2548
